# Supplementary material for: Engineering thin 3D Li-composite foil negative electrodes with high mechanical toughness
Source: Nat Commun. 2026 Feb 4;17:2345. doi: 10.1038/s41467-026-69155-z (PMC12979835; doi:10.1038/s41467-026-69155-z)
Supplement: Supplementary file 1 — Supplementary Information [file 41467_2026_69155_MOESM1_ESM.pdf]

Supplementary Information

Engineering thin 3D Li-composite foil negative electrodes with high mechanical toughness

Yu-Hao Wang [1, 2, #], Shuang-Jie Tan [1, #], Chao-Hui Zhang [1], Jun-Chen Guo [1], Xiao-Xi Luo [1], Ruo-Xi Jin [1, 2], Lin-Bo Huang [1], Xiao-Chuan Su [1, 2], Chen Li [1, 2], Xu-Sheng Zhang [1], Xing Zhang [1], Sen Xin [1, 2], Rui Wen [1, 2], Juan Zhang [1], Yu-Guo Guo* [1, 2]*

Y.-H. Wang, Dr. S.-J. Tan, Dr. C.-H. Zhang, Dr. J.-C. Guo, X.-X. Luo, R.-X. Jin, Dr. L.-B. Huang, X.-C. Su, C. Li, Dr. X.-S. Zhang, Dr. X. Zhang, Prof. S. Xin, Prof. R. Wen, Prof. J. Zhang, Prof. Y.-G. Guo
[1]CAS Key Laboratory of Molecular Nanostructure and Nanotechnology/ Institute of Chemistry, Chinese Academy of Sciences (CAS), Beijing 100190, P. R. China
E-mail: zhangjuan120@iccas.ac.cn; ygguo@iccas.ac.cn

Y.-H. Wang, R.-X. Jin, X.-C. Su, C. Li, Prof. S. Xin, Prof. R. Wen, Prof. Y.-G. Guo
[2]School of Chemical Sciences, University of Chinese Academy of Sciences (UCAS), Beijing 100049, P. R. China

[#]These authors contributed equally: Yu-Hao Wang, Shuang-Jie Tan.

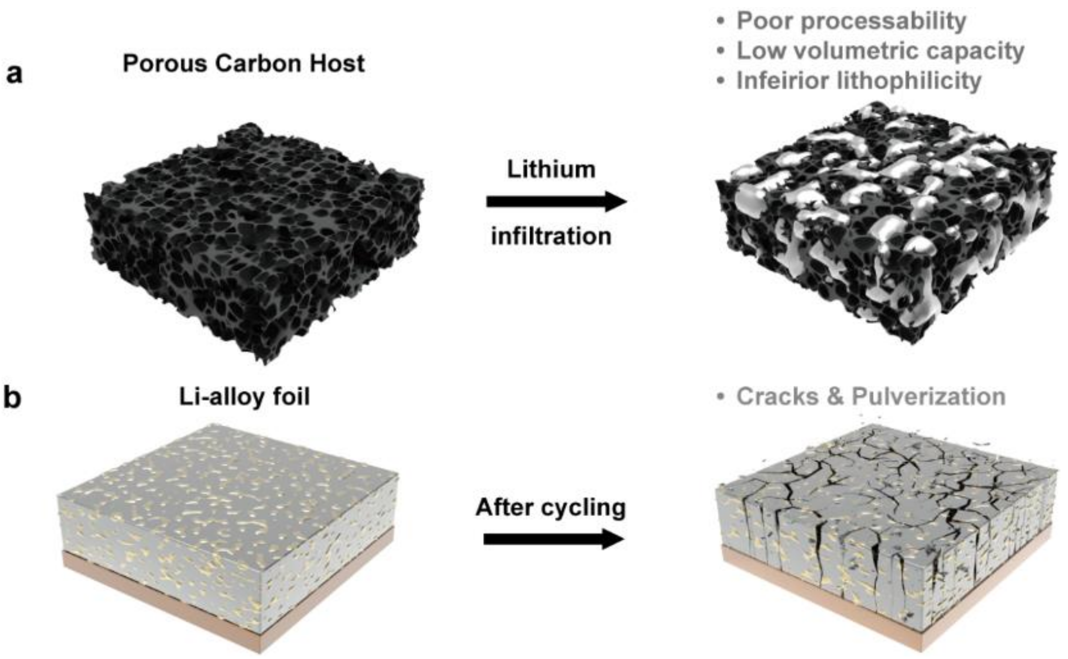

Figure S1. Schematic of challenges in 3D composite lithium negative electrodes. Schematic illustrating the challenges associated with a, a three-dimensional (3D) porous carbon-based composite lithium negative electrode, and b, a 3D metallic-based composite lithium negative electrode.

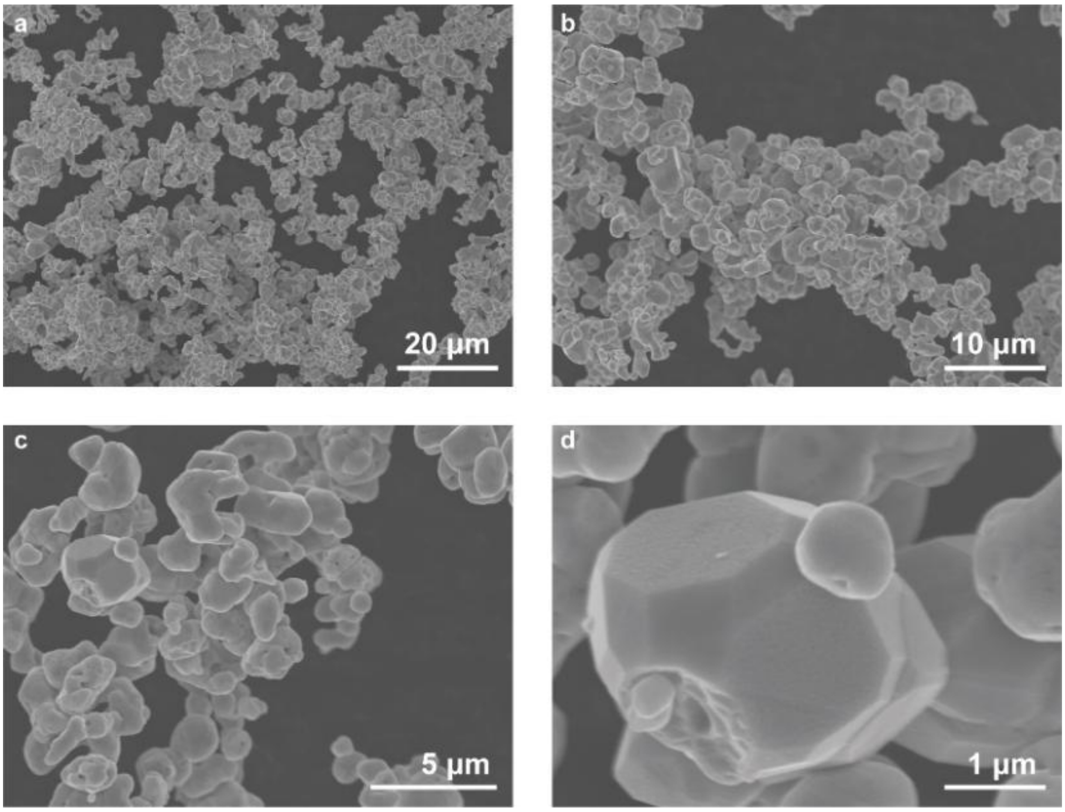

Figure S2. SEM images of Zn_3N_2 at different magnifications. a-d, representative images showing the morphology of Zn_3N_2 at progressively increasing length scales.

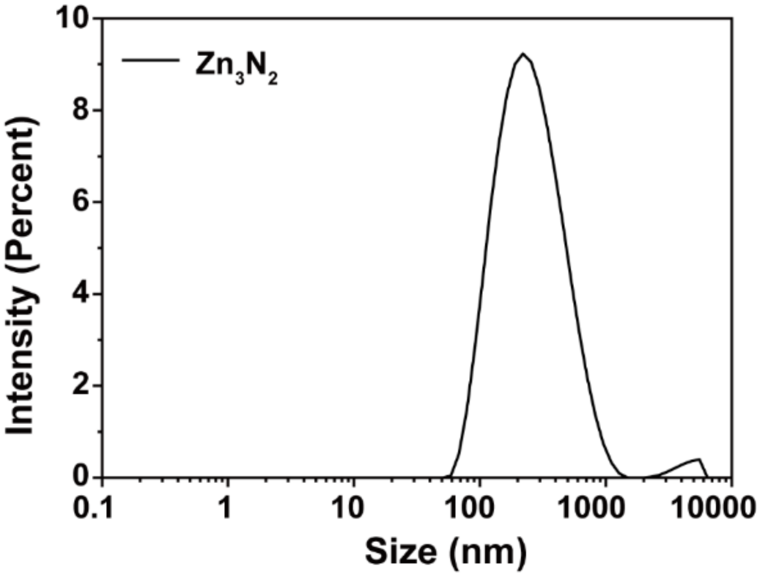

Figure S3. Particle size distribution of Zn_3N_2.

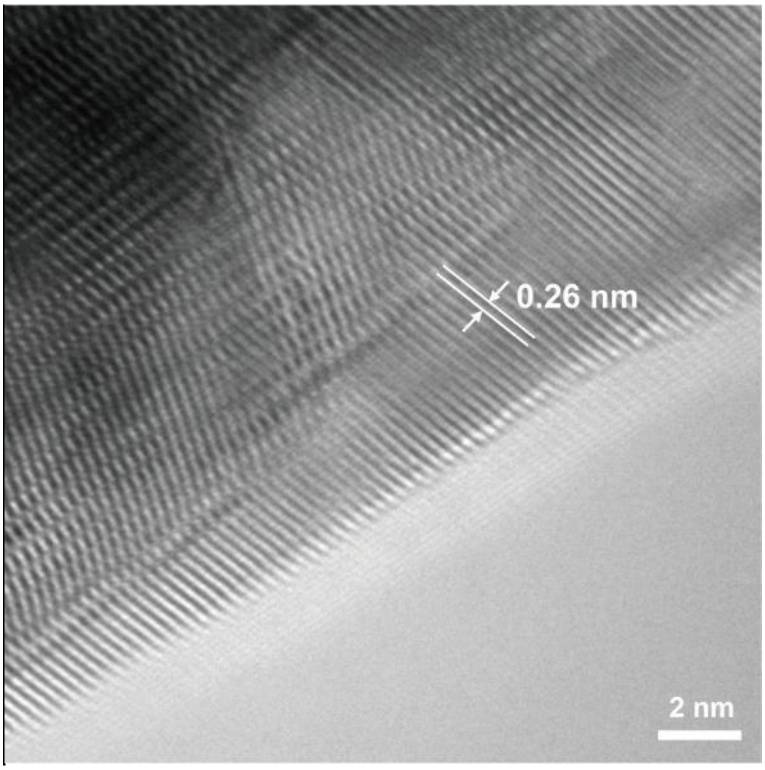

Figure S4. TEM image of Zn_3N_2.

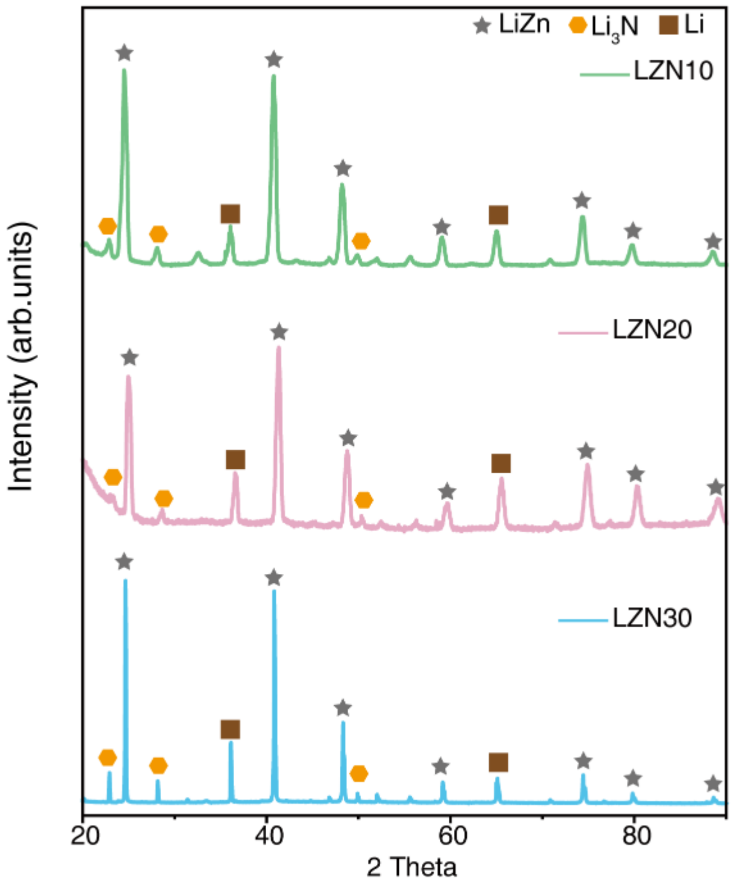

Figure S5. XRD pattern of Li-Zn_3N_2 composites with different mass ratios. The LZN10, LZN20, LZN30 compounds are produced by melting lithium metal and Zn_3N_2 in molar ratios of 30:1, 60:1 and 90:1, respectively.

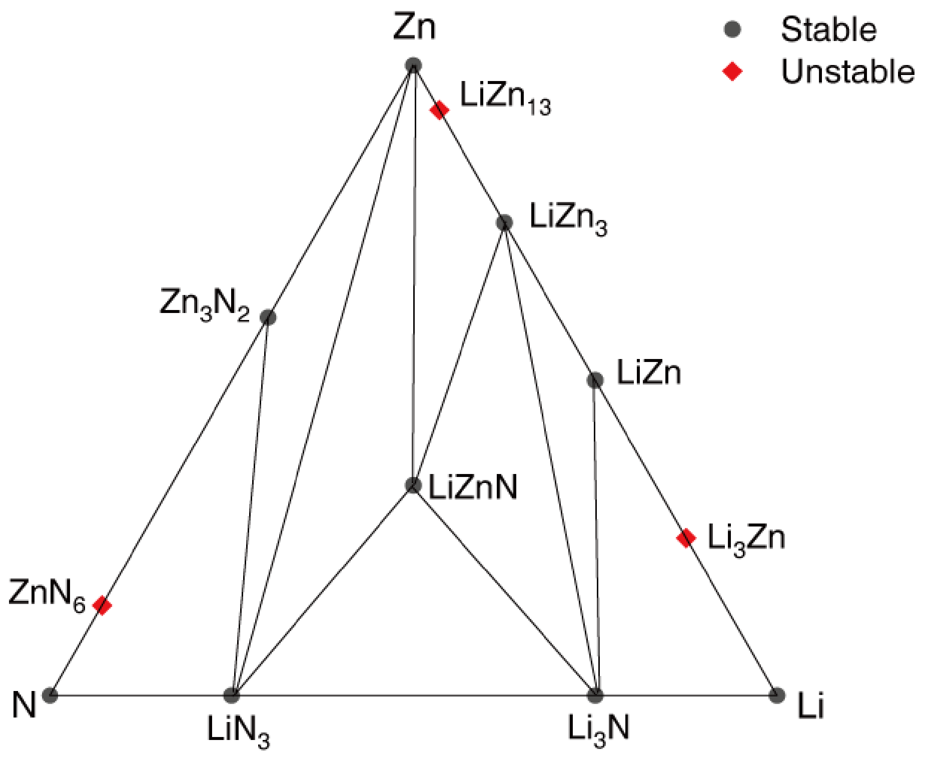

Figure S6. Ternary phase diagram of Li-Zn-N. According to the ternary phase diagram, the Li: Zn: N molar ratio of 60:3:2 corresponds to the stabilized components of Li, LiZn and Li_3N, which is consistent with the XRD characterization.

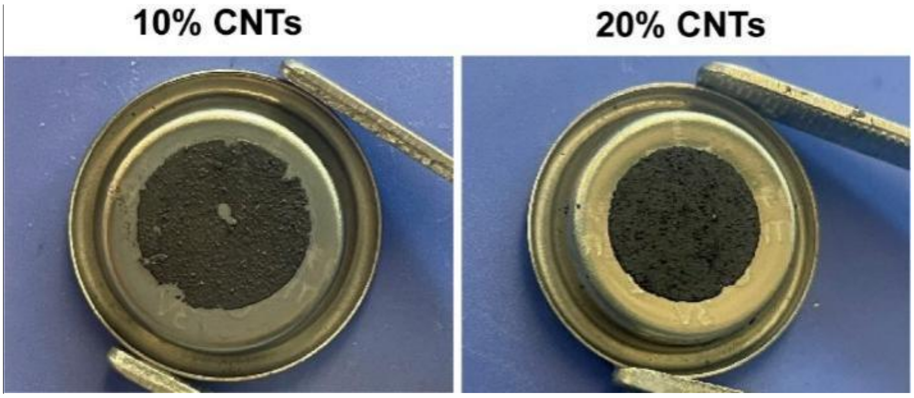

Figure S7. Digital photographs of fully delithiated LZNC with different CNTs mass ratios after Li stripping at 0.5 mA cm^{-2} with a cutoff voltage of 1 V vs Li^+/Li.

Note S1: At 10% wt CNTs incorporation, optical images reveal that the negative electrode still collapses after 75% lithium stripping, whereas 20% wt CNTs incorporation maintains good integrity and continuity of the negative electrode. Therefore, the optimal mass ratio of CNTs was determined to be 20%.

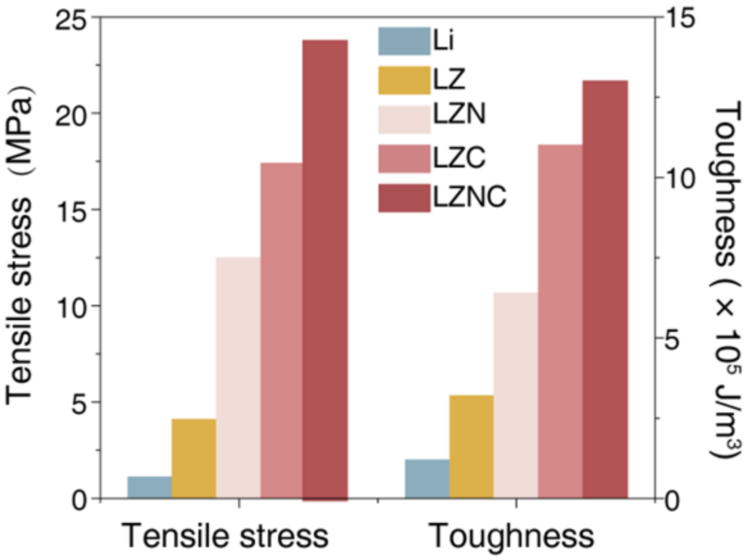

Figure S8. Columnar Comparison Chart of Tensile Strength and Toughness for Li, LZ, LZN, LZC, and LZNC.

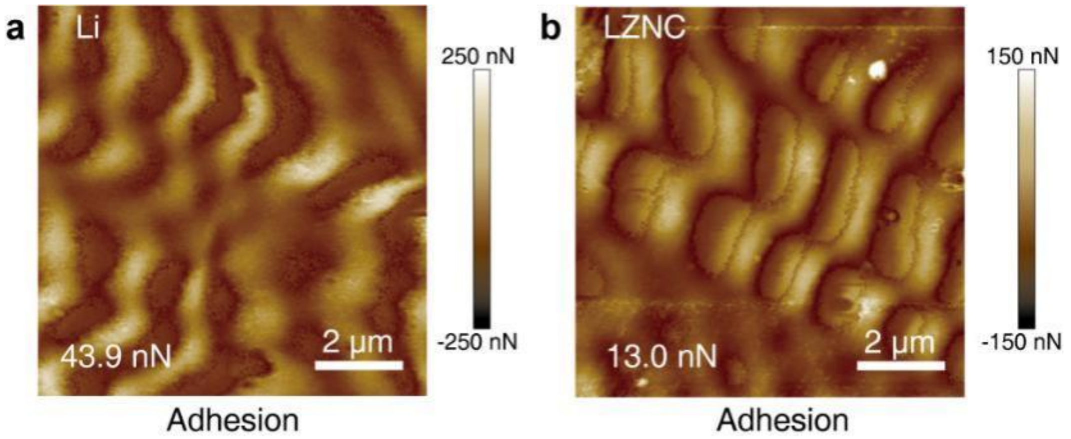

Figure S9. AFM adhesion of bare Li and LZNC surfaces. AFM adhesion maps of a) bare Li and b) LZNC surfaces.

Note S2. The results of the AFM adhesion test (Supplementary Fig. 8) reveal that the adhesion of LZNC (13.0 nN) is significantly lower than that of lithium foil (43.9 nN), indicating improved machinability compared to pure Li.

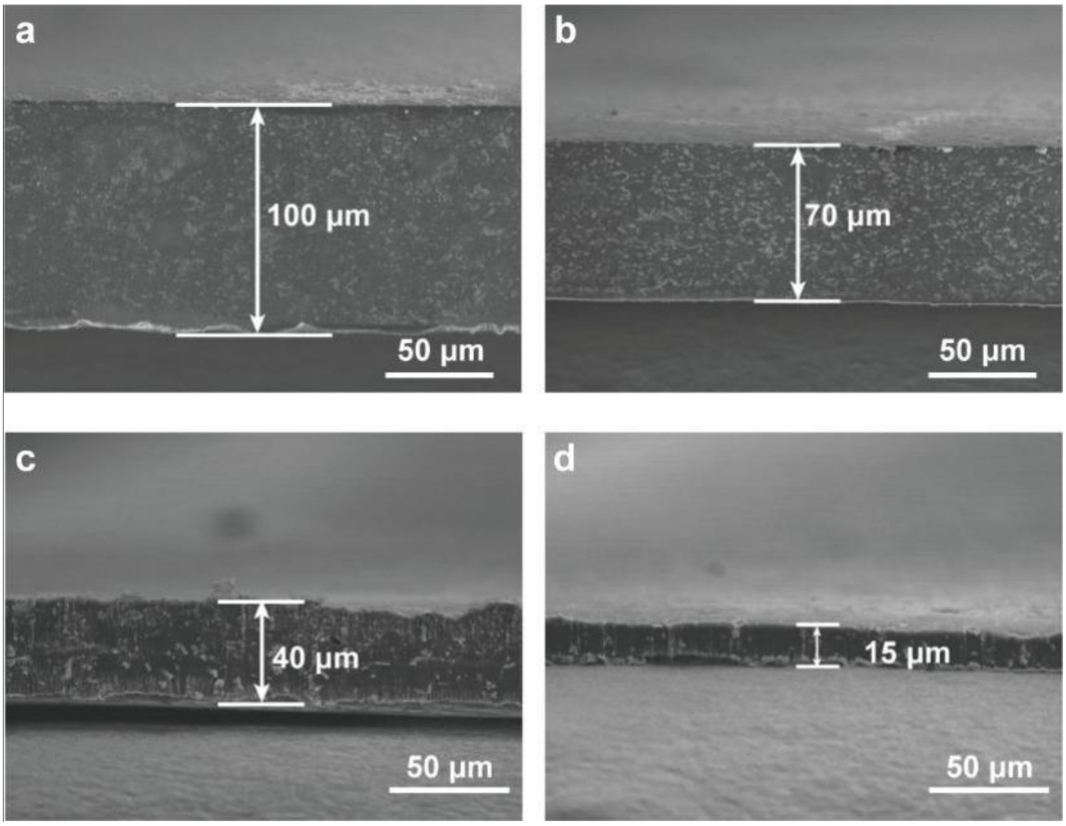

Figure S10. Cross-sectional SEM characterization of LZNC foils. Cross-sectional scanning electron microscopy (SEM) images of LZNC foils with thicknesses of a) 100 μm; b) 70 μm; c) 40 μm; and d) 15 μm.

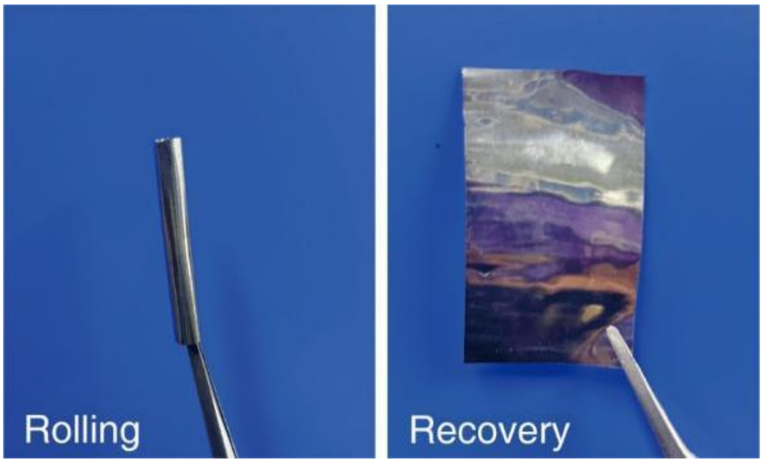

Figure S11. Digital photographs of the 50 μm-thick LZNC composite foil under the flexibility test.

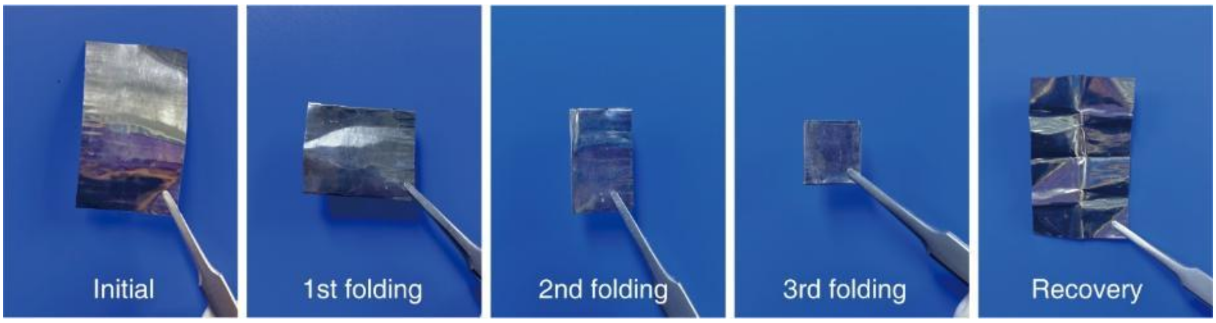

Figure S12. Digital photographs of the 50 μm-thick LZNC foil during the folding test.

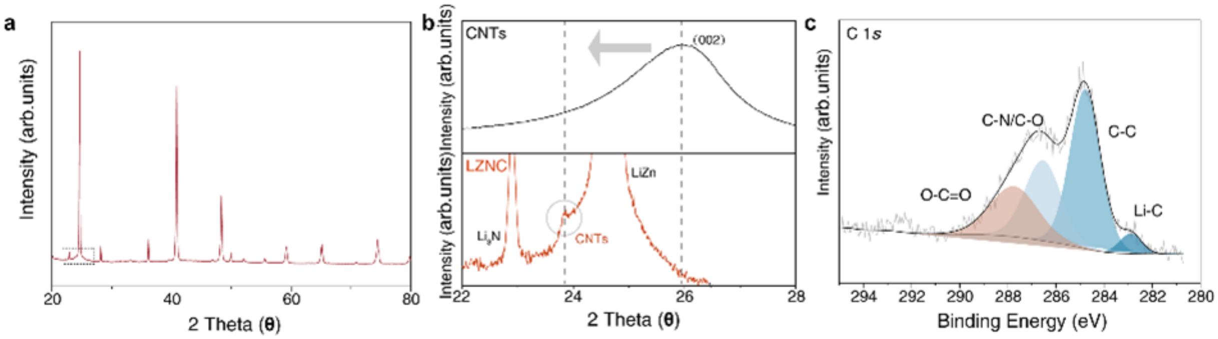

Figure S13. Evidence of carbon lithiation in LZNC. a) XRD pattern of LZNC. b) XRD patterns of pristine CNTs and LZNC, showing an enlarged view of the region corresponding to the dashed rectangle in (a). c) XPS spectra of C 1s of the LZNC composite.

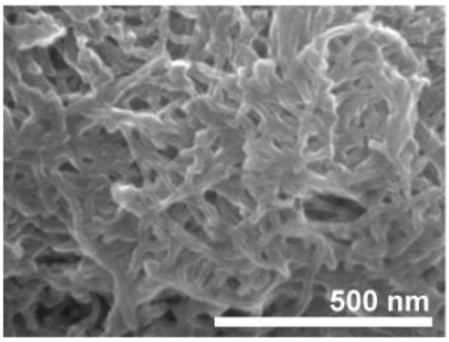

Figure S14. SEM image of LZNC after complete de-lithiation after Li stripping at 0.5 mA cm^{-2} with a cutoff voltage of 1 V vs Li^{+}/Li.

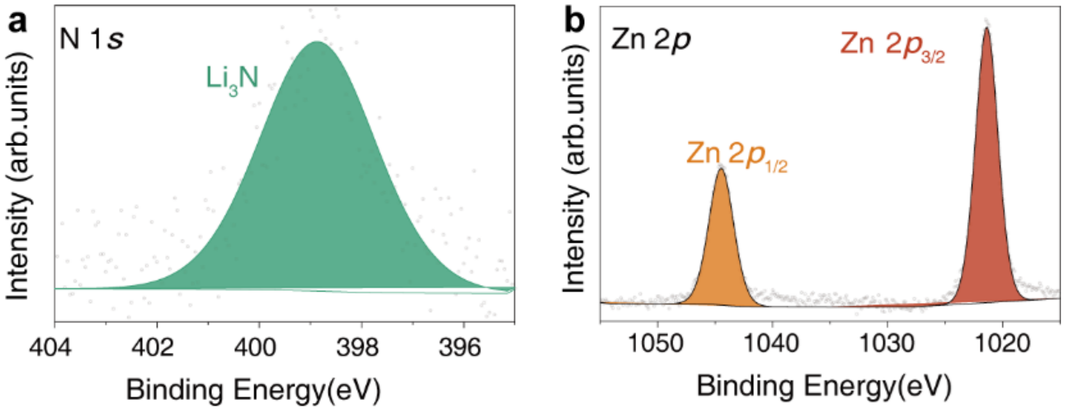

Figure S15. High-resolution XPS spectra of the LZNC composite. a) N 1s spectrum; b) Zn 2p spectrum.

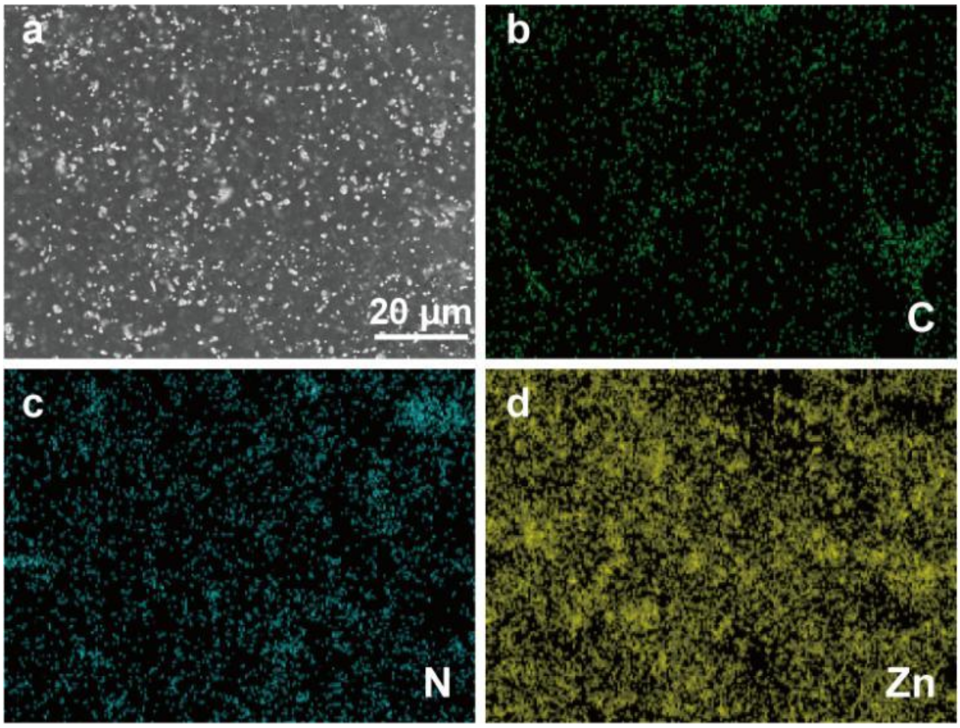

Figure S16. Top-view SEM and elemental mapping of LZNC. (a) Top-view SEM image of the LZNC composite and the corresponding element mapping for b) C, c) N, and d) Zn.

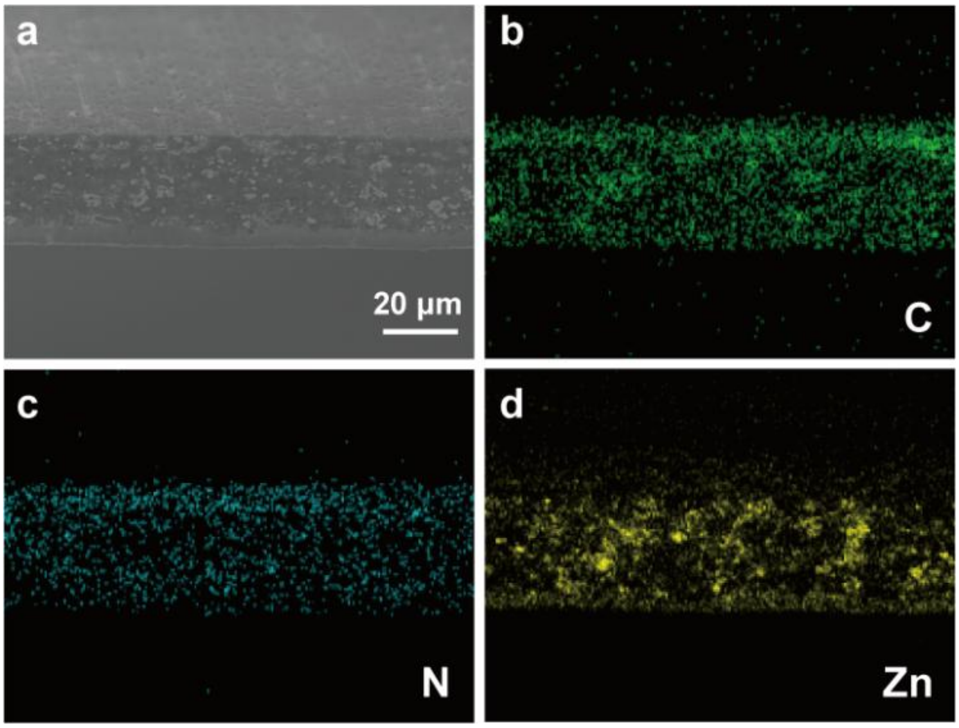

Figure S17. Cross-view SEM and elemental mapping of LZNC. (a) cross-section SEM image of the LZNC composite and the corresponding element mapping for b) C, c) N, and d) Zn.

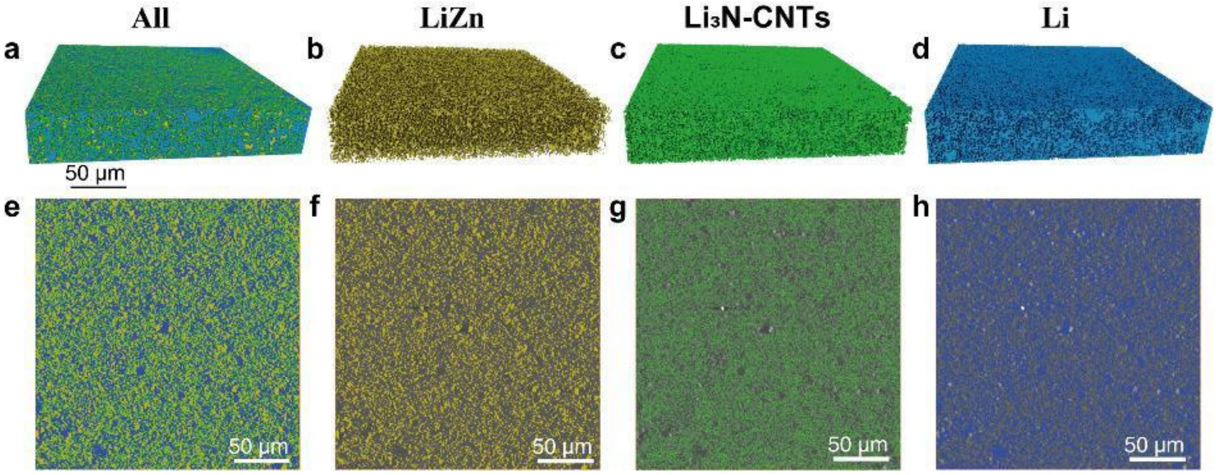

Figure S18. 3D microstructural analysis of LZNC. a-d) 3D microstructural analysis of LZNC and e-h) x-y slices detected by x-ray microscope (XRM).

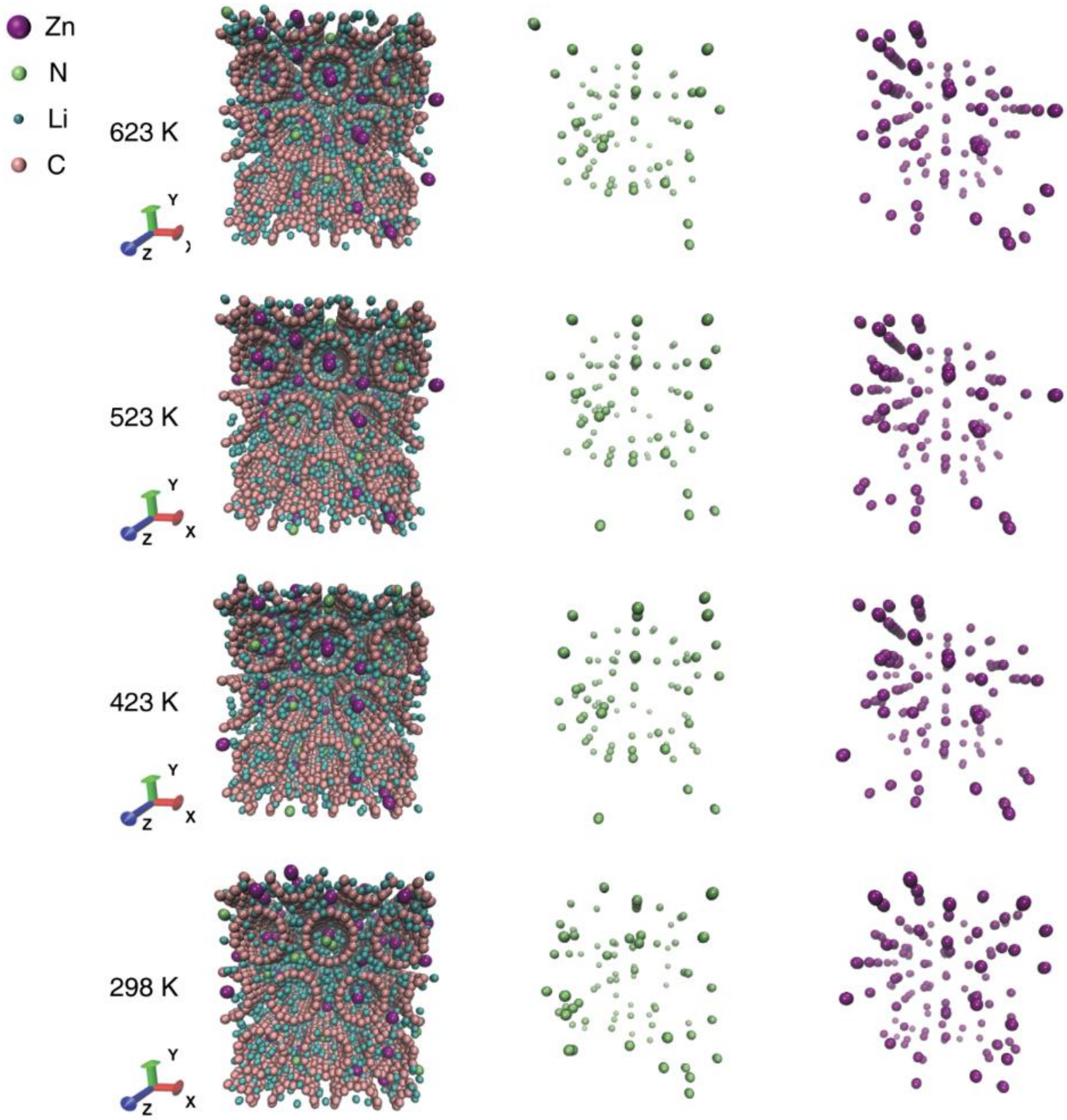

Figure S19. Schematic of MD simulation of LZNC during annealing process.

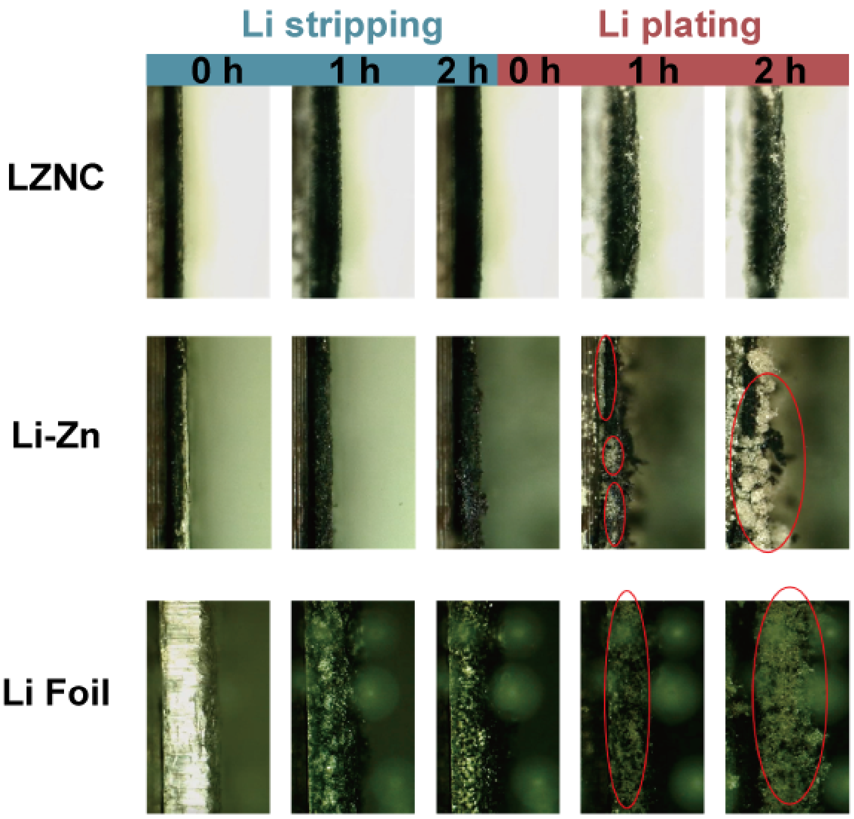

Figure S20. The in situ optical observations of Li stripping/plating processes on Li, Li-Zn and LZNC electrodes at a current density of 1 mA cm^{-2}.

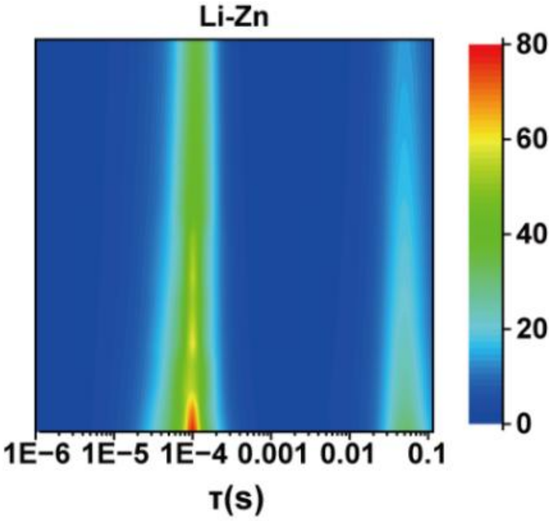

Figure S21. In situ DRT analysis of the Li stripping and plating processes of Li-Zn negative electrode at 0.5 mA cm^{-2}.

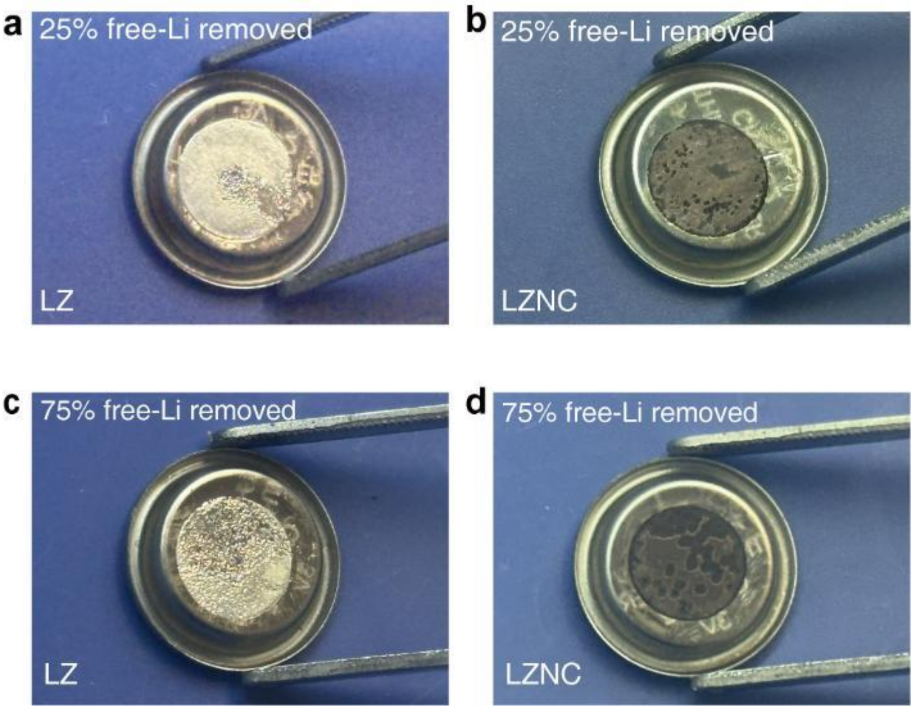

Figure S22. Digital photograph of Li-Zn and LZNC stripped free Li at 0.5 mA cm^{-2}. a) Li-Zn after 25% Li stripped; b) LZNC after 25% Li stripped; c) Li-Zn after 75% Li stripped; d) LZNC after 75% Li stripped.

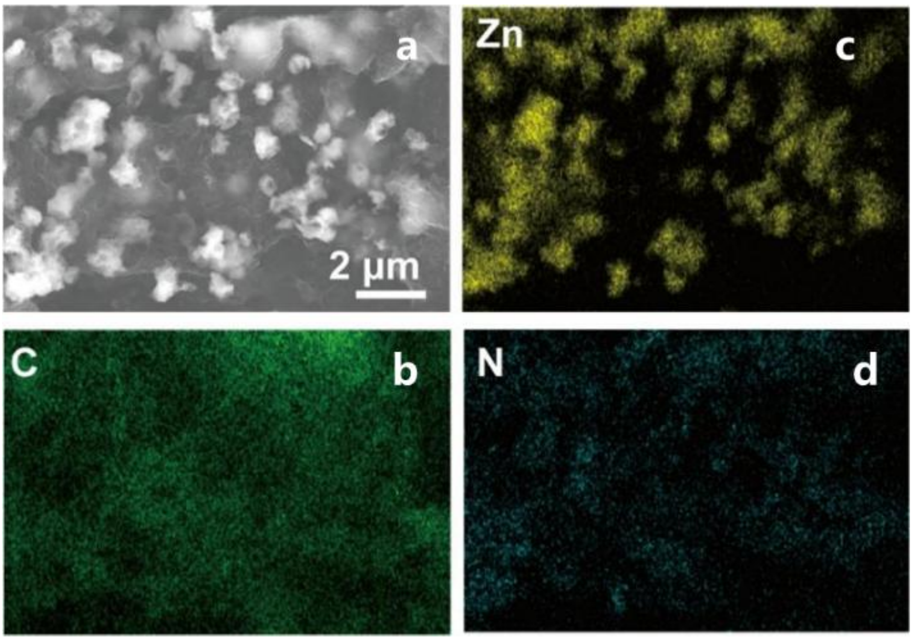

Figure S23. SEM and elemental mapping of LZNC after Li stripping. a) SEM image and b-d) corresponding EDS mapping of LZNC after Li stripping at 0.5 mA cm^{-2} with a cutoff voltage of 1 V vs Li^{+}/Li.

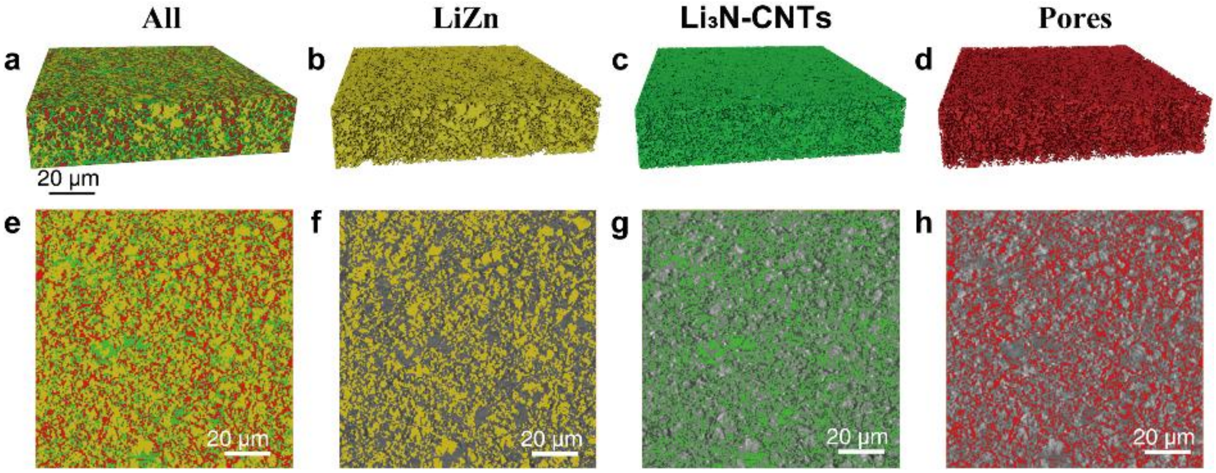

Figure S24. 3D microstructural analysis of LZNC after free Li stripped. a-d) 3D microstructural analysis of LZNC after Li stripping at 0.5 mA cm^{-2} with a cutoff voltage of 1 V vs Li^+/Li and e-h) x-y slices detected by XRM.

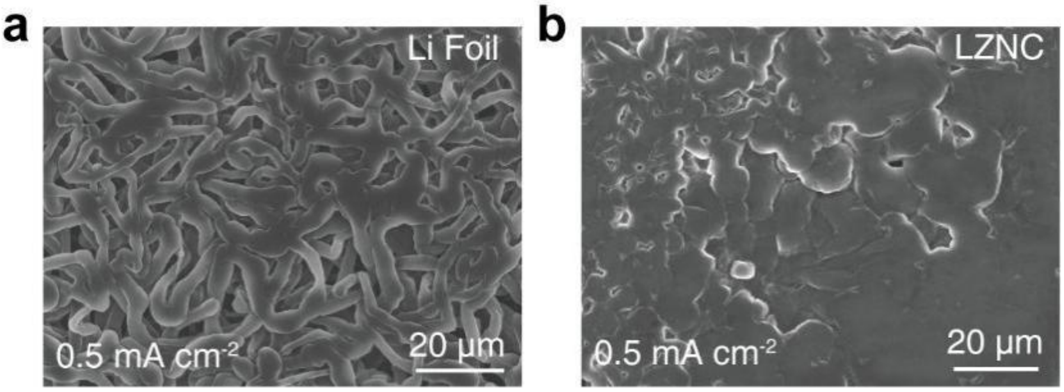

Figure S25. Top-view SEM images after 25% delithiated and first plating. a) bare Li and b) LZNC after the first plating under under 0.5 mA cm^{-2} with a capacity of 6 mA h cm^{-2}.

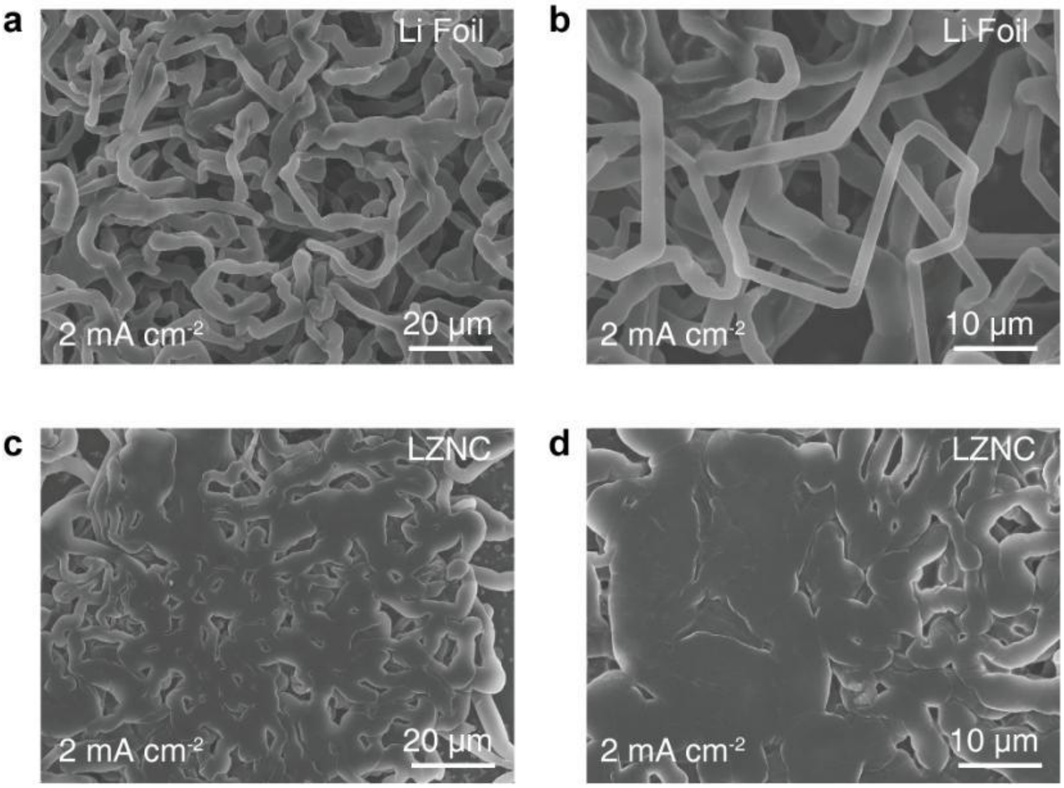

Figure S26. Top-view SEM images after 25% delithiated and first plating at 2 mA cm^{-2}. Top-view SEM images of 25% delithiated a, b) bare Li and c, d) LZNC after the first plating under 2 mA cm^{-2} with a capacity of 6 mA h cm^{-2}.

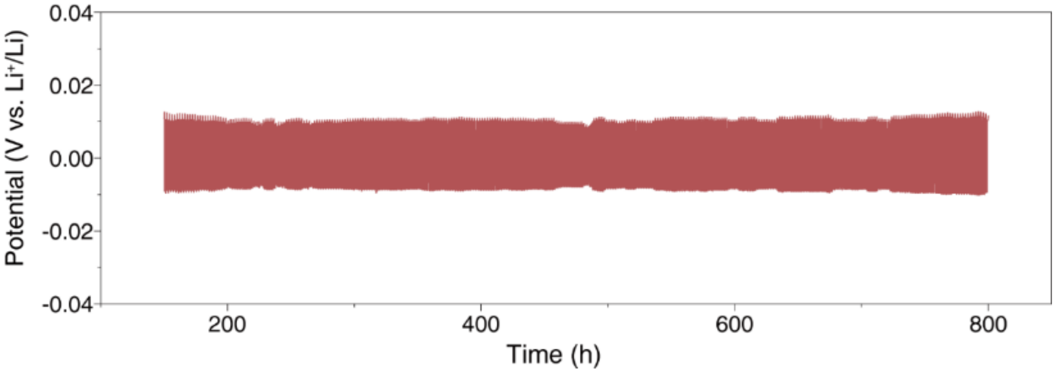

Figure S27. Voltage profiles of LZNC symmetric cell under 1 mA cm^{-2}- 1 mA h cm^{-2} conditions.

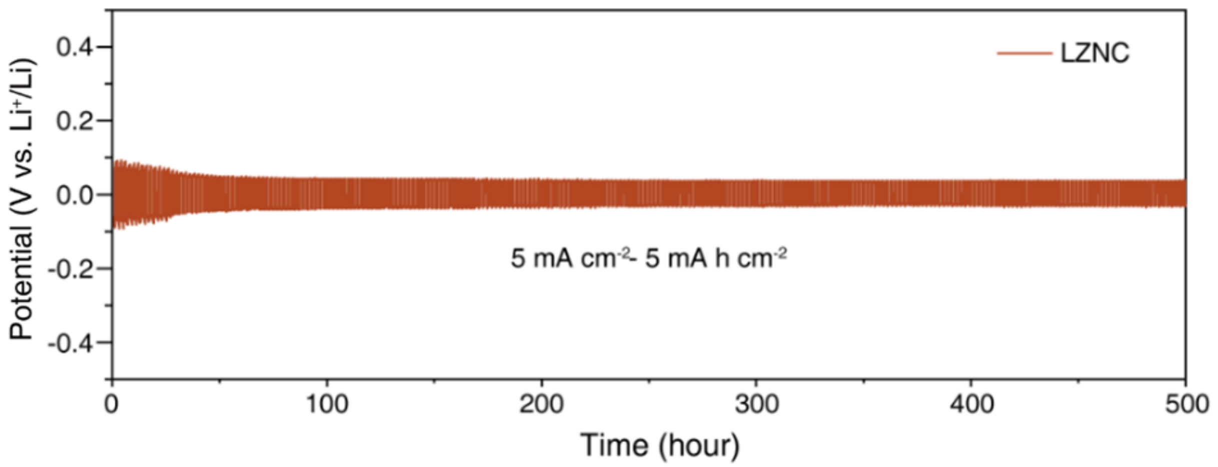

Figure S28. Voltage profile of symmetric cell with bare Li and LZNC under 5 mA cm^{-2} with a capacity of 5 mA h cm^{-2}.

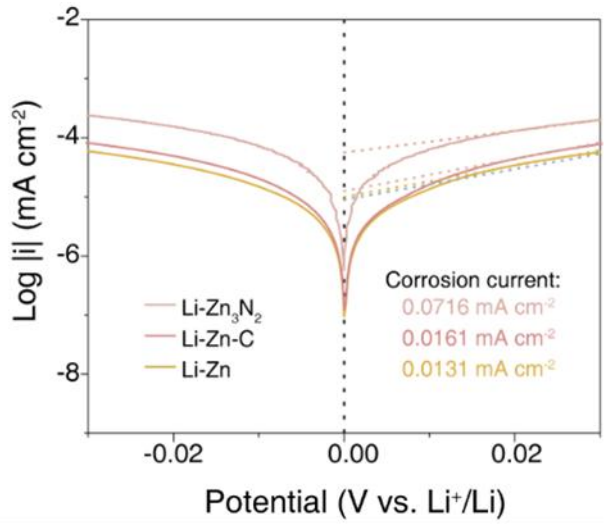

Figure S29. Tafel plots of bare Li-Zn, LZN and LZC symmetric cells.

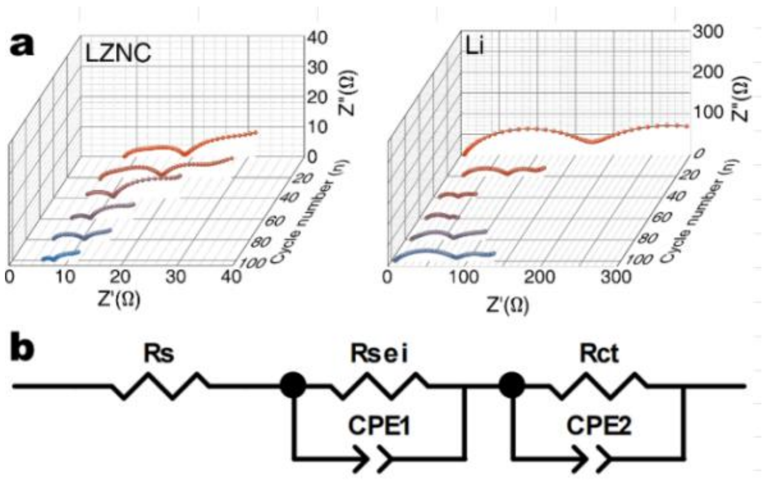

Figure S30. EIS and equivalent circuit of LZNC. a) Nyquist plots of LZNC and Li foil at different Li plating/stripping stages under 1 mA cm^{-2} and 1 mA h cm^{-2}. b) Equivalent circuit diagram of symmetrical cell.

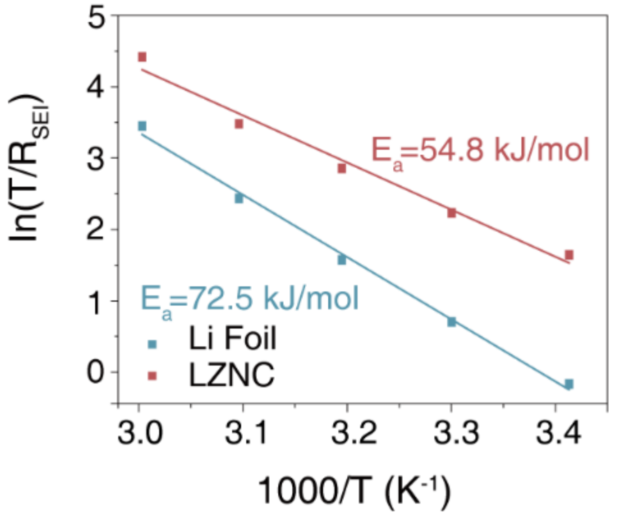

Figure S31. Arrhenius behavior of the resistant, where the activation energy (E_a) of the bare Li and LZNC was obtained.

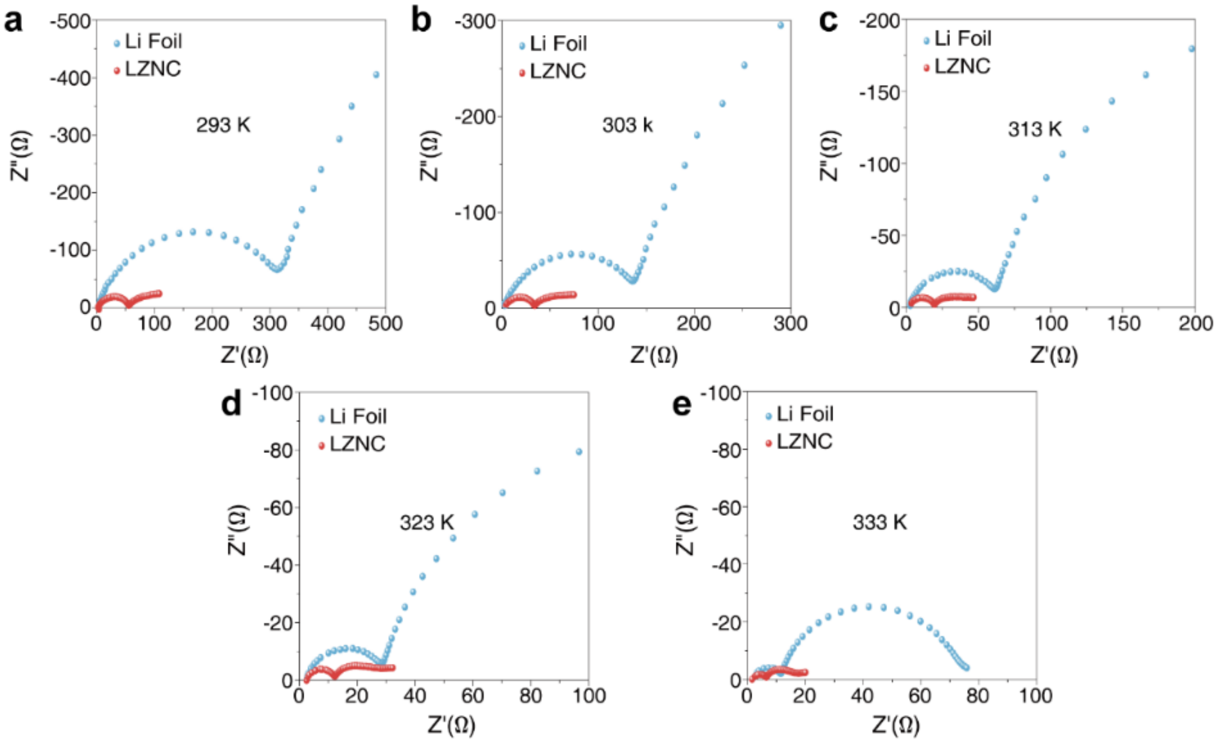

Figure S32. Temperature-dependent Nyquist plots of symmetric cells. Nyquist plots of symmetric cells with Li foil and LZNC, respectively at a) 293K, b) 303K, c) 313K, d) 323K, e) 333K.

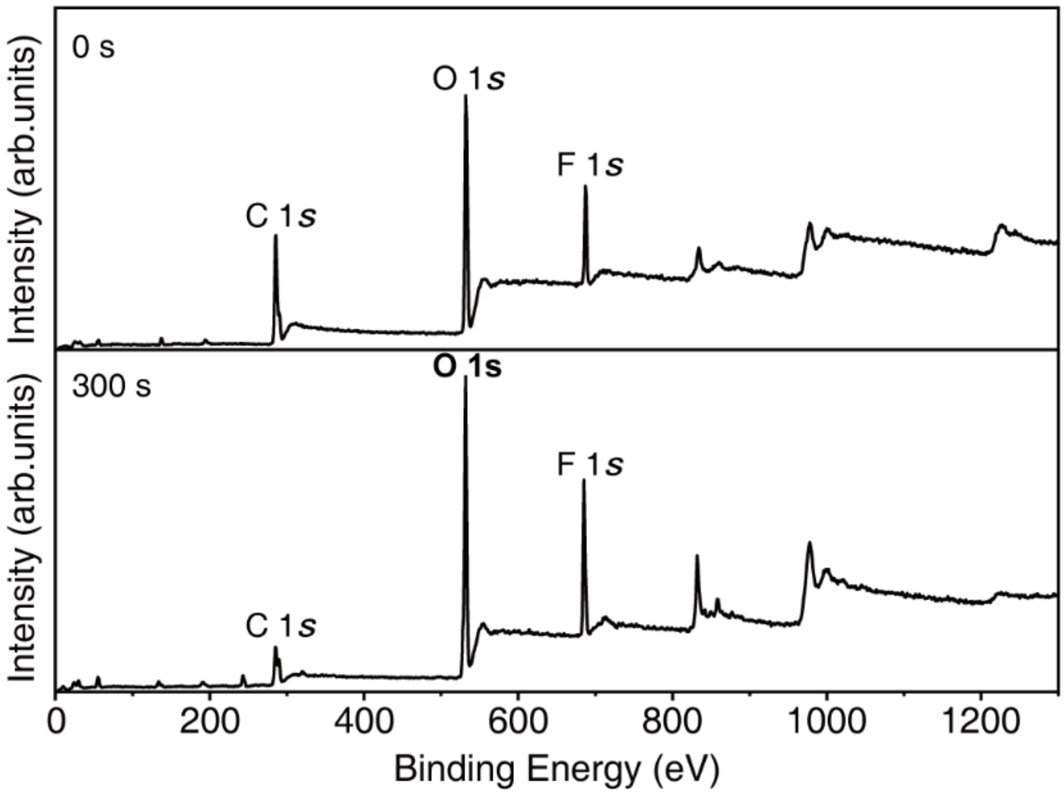

Figure S33. XPS full-scan survey spectra of bare Li after Li plated/stripped over 100 h at 1 mA cm^{-2} with a capacity of 1 mA h cm^{-2}.

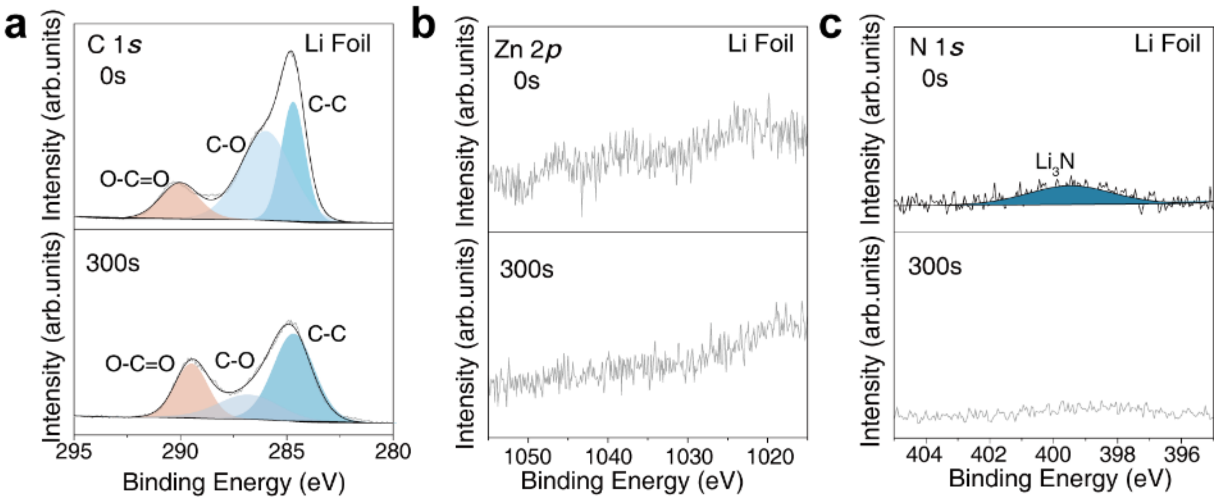

Figure S34. XPS depth profile of bare Li after cycling. XPS depth profile of a) C *1s*, b) Zn *2p*, c) N *1s* for the bare Li negative electrode after 100 h at 1 mA cm^{-2} with a capacity of 1 mA h cm^{-2}.

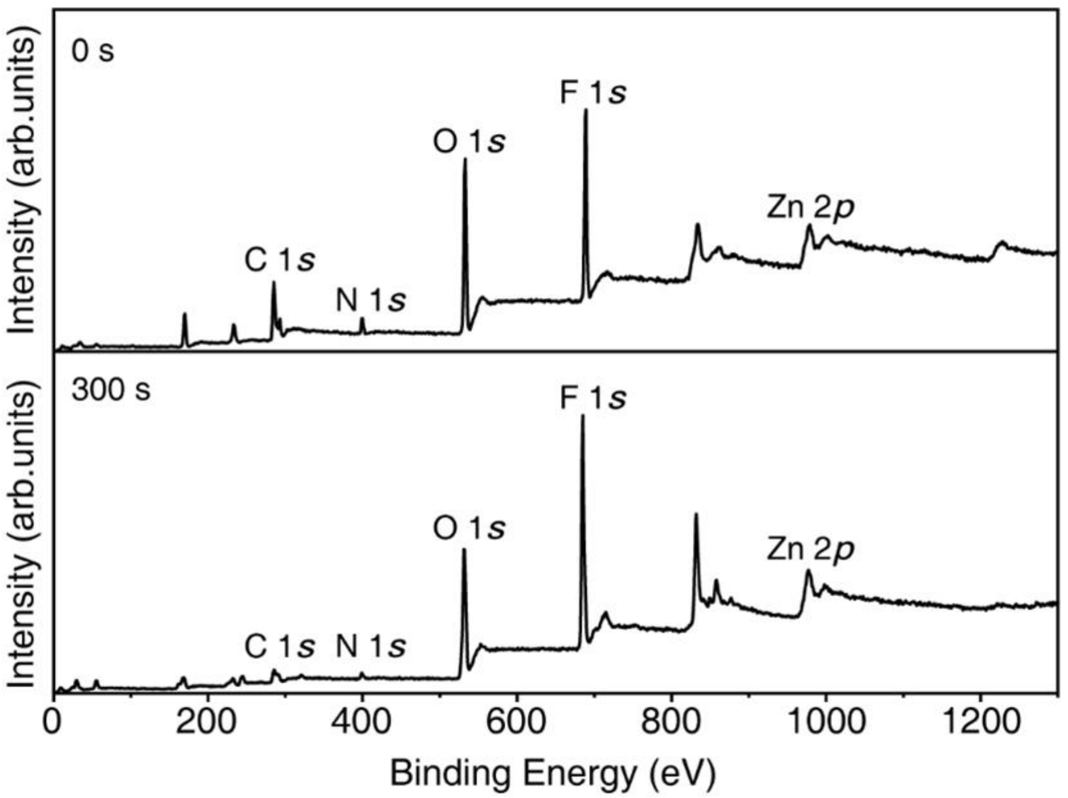

Figure S35. XPS full-scan survey spectra of LZNC after 100 h at 1 mA cm^{-2} with a capacity of 1 mA h cm^{-2}. The signals of N and Zn are much larger than those of C.

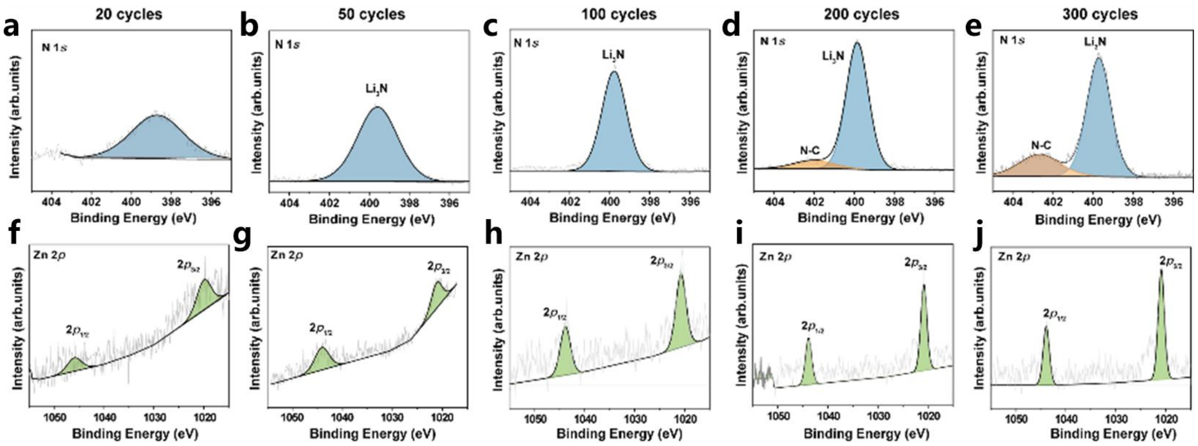

Figure S36. XPS profiles of N *1s* and Zn *2p* of LZNC at different Li plating/stripping cycles. XPS profiles of LZNC during lithium plating and stripping under 1 mA cm^{-2} with a capacity of 1 mA h cm^{-2}, showing both the a-e) N *1s* spectra and the corresponding f-j) Zn *2p* spectra at 20, 50, 100, 200, and 300 cycles.

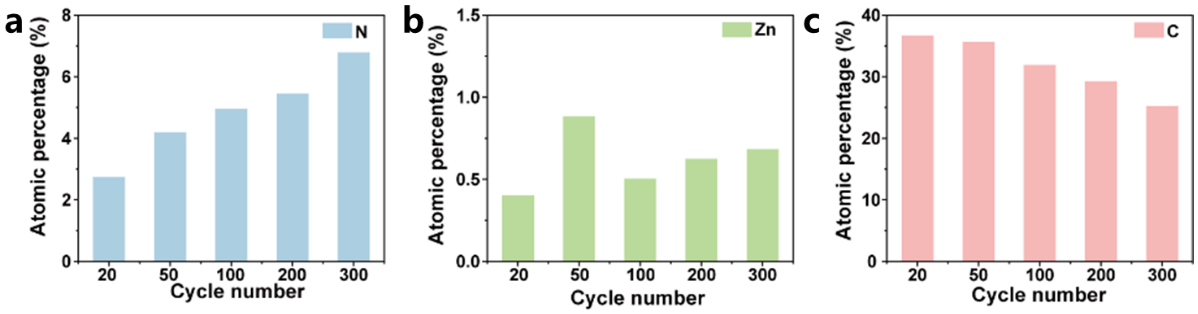

Figure S37. Surface atomic percentages of LZNC at different Li plating/stripping stages. Atomic percentages of LZNC negative electrodes were measured by XPS under 1 mA cm^{-2} with a capacity of 1 mA h cm^{-2}, showing the a) N, b) Zn, and c) C.

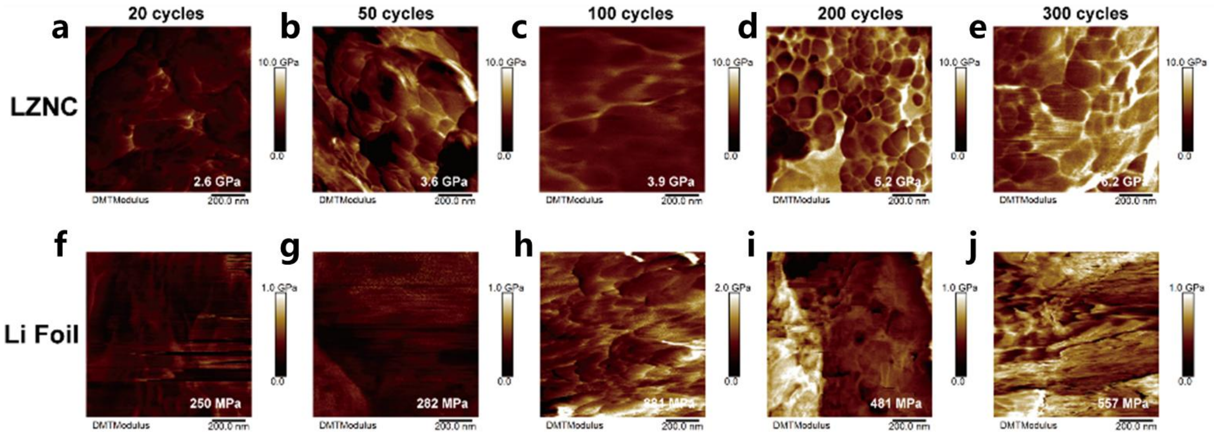

Figure S38. AFM modulus of LZNC and Li foil at different Li plating/stripping stages. AFM modulus of LZNC and Li foil under 1 mA cm^{-2} with a capacity of 1 mA h cm^{-2}, showing the a-e) LZNC modulus after 20, 50, 100, 200, and 300 cycles and the f-j) Li foil modulus for the same cycles.

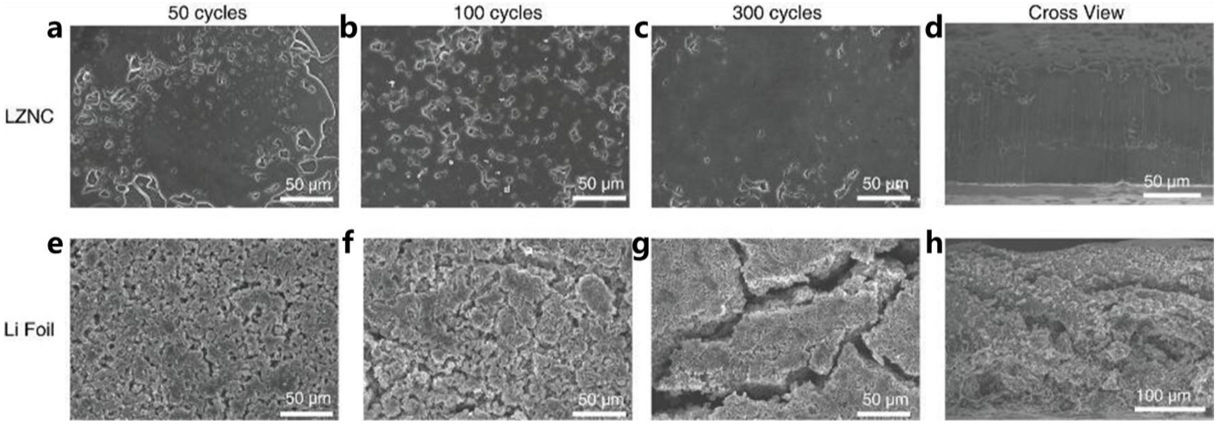

Figure S39. **SEM images of LZNC and Li foil surfaces at different Li plating/stripping stages.** SEM images of LZNC and Li foil under 1 mA cm^{-2} with a capacity of 1 mA h cm^{-2}, showing the a-c) top-view LZNC surfaces after 50, 100, and 300 cycles, the d) cross-section of LZNC, the e-g) top-view Li foil surfaces for 50, 100, and 300 cycles, and the h) cross-section of Li foil.

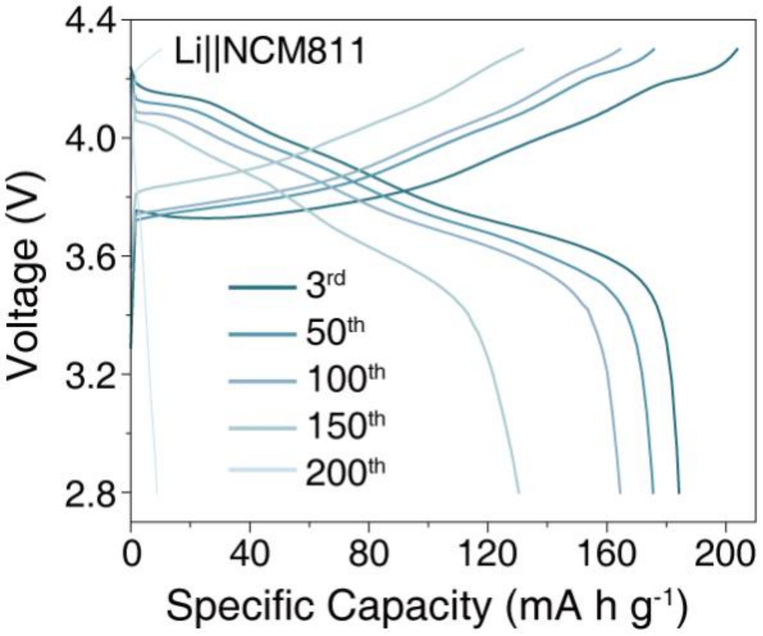

Figure S40. The corresponding voltage profiles of Li||NCM811 full cells.

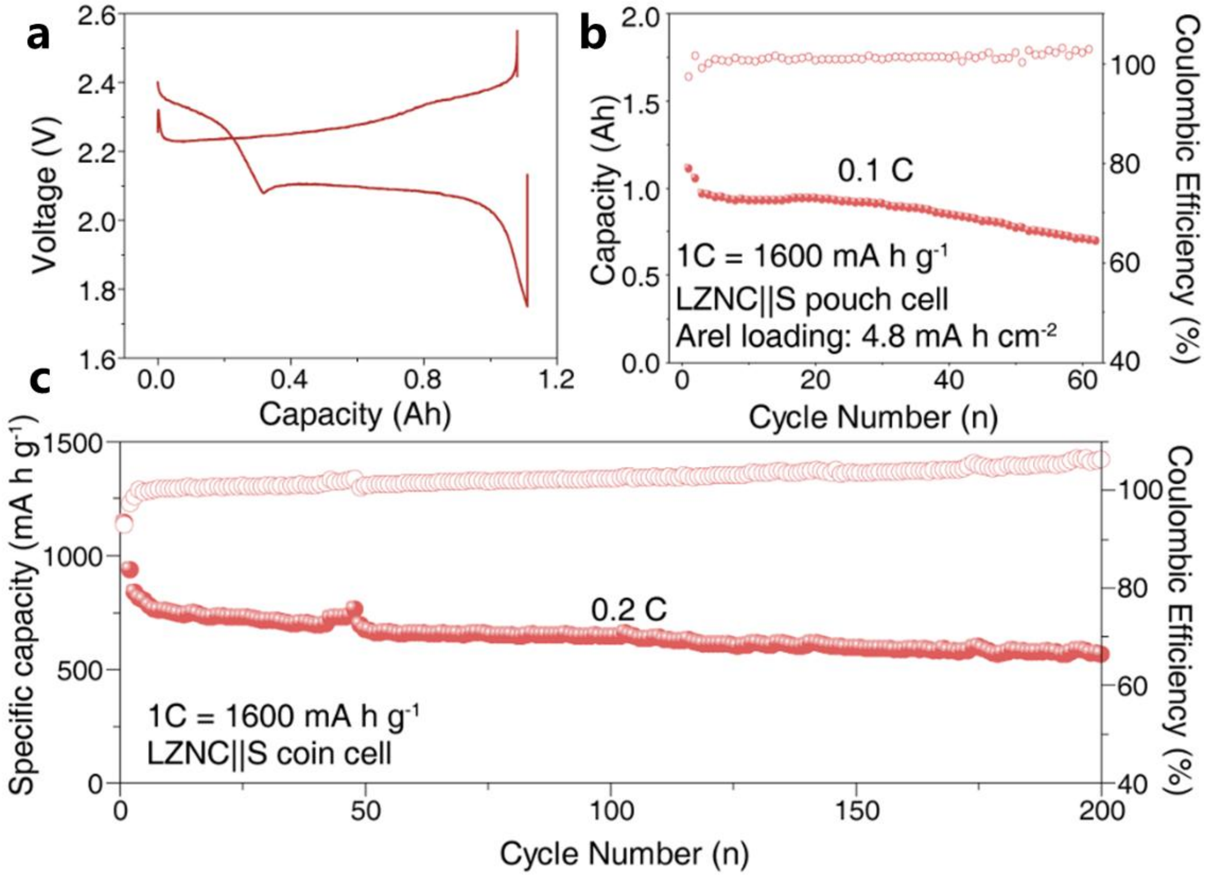

Figure S41. Cycling performance and corresponding voltage profiles of LZNC||S coin-type and pouch cells (1 C = 1600 mA g⁻¹). a) first-cycle voltage profile of the pouch cell; b) cycling curve of the pouch cell; c) cycling curve of the coin-type cell.

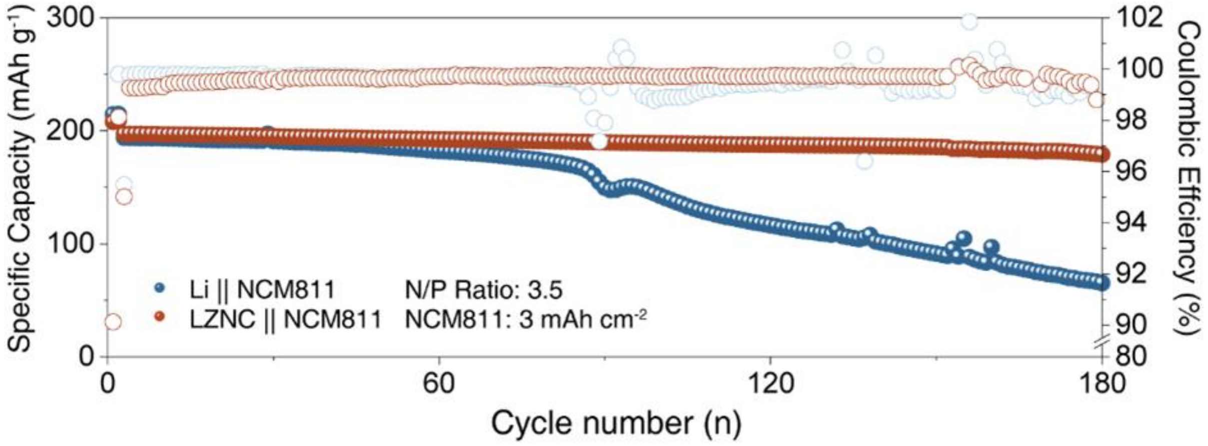

Figure S42. Cycling performance of high-loaded NCM811 (3 mA h cm^{-2}) full cells with LZNC negative electrodes under 0.3 C.

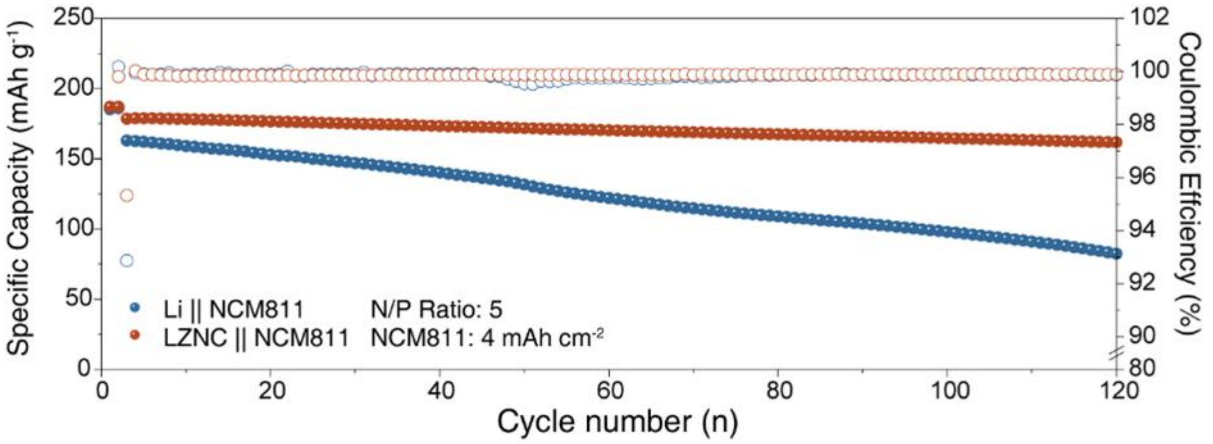

Figure S43. Cycling performance of high-loaded NCM811 (4 mA h cm^{-2}) full cells with 100 μm LZNC negative electrodes under 0.3 C.

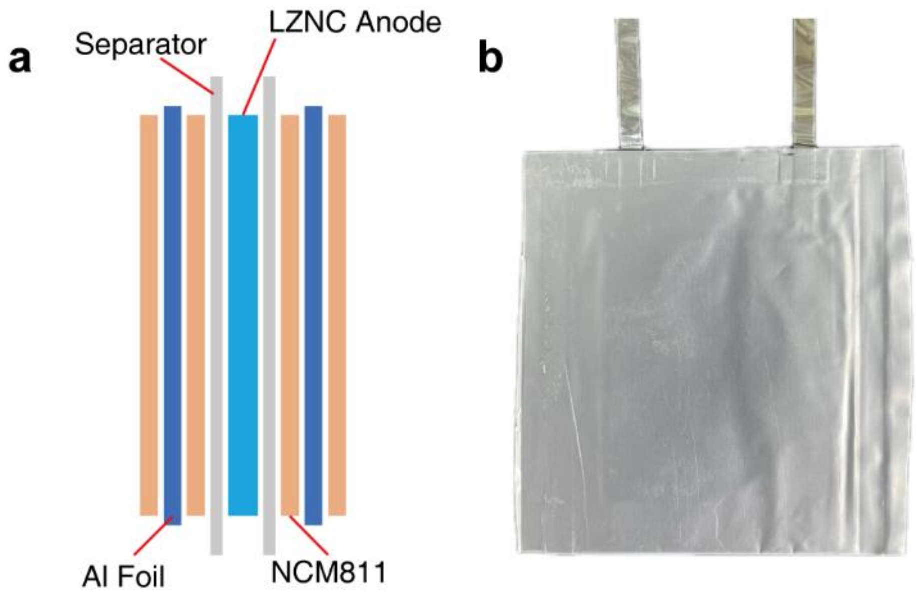

F**igure S44. Illustration and photograph of LZNC||NCM811 pouch cell.** a) Schematic illustrating and b) digital photograph of LZNC||NCM811 pouch cell.

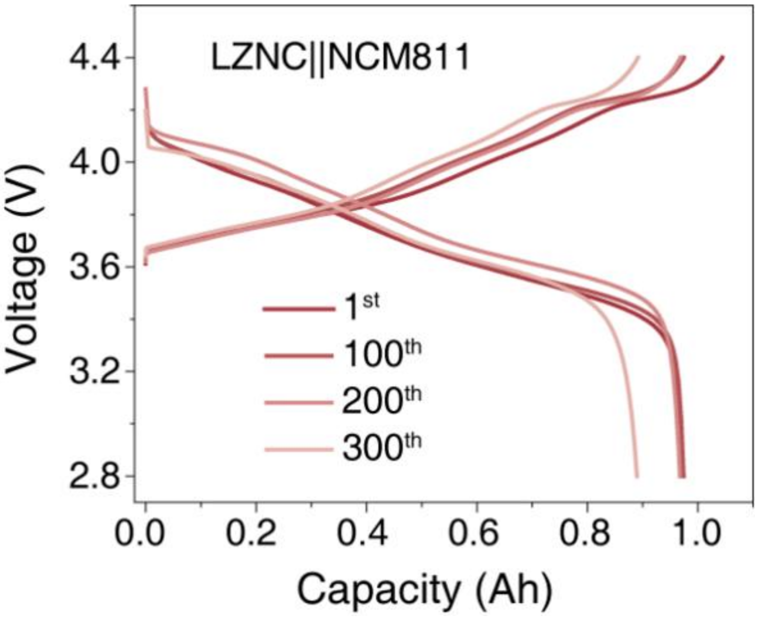

Figure S45. Voltage profiles of the LZNC||NCM811 pouch cell.

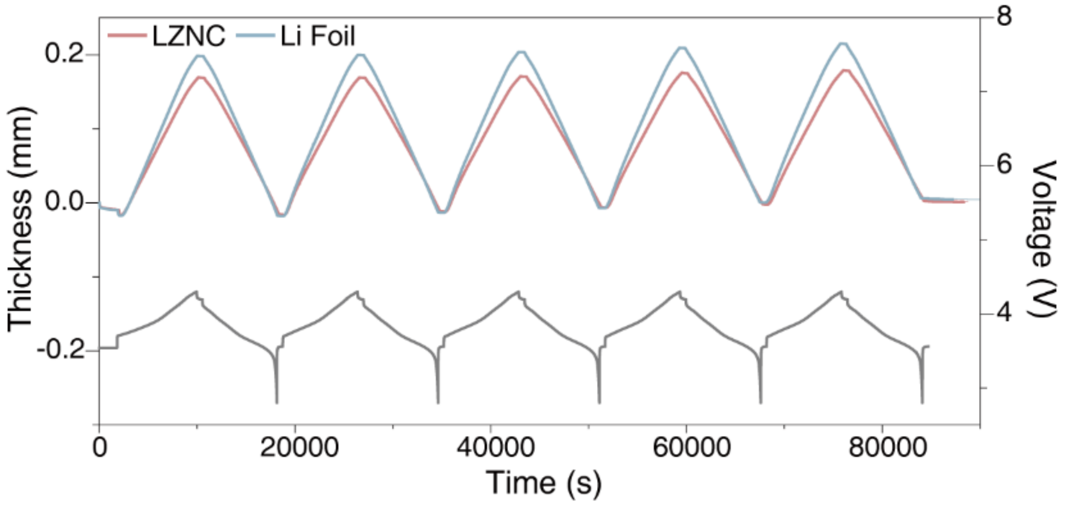

Figure S46. Thickness variation of LZNC||NCM811 and Li||NCM811 pouch cell during 0.5 C charge-discharge cycling under a constant pressure of 0.2 MPa.

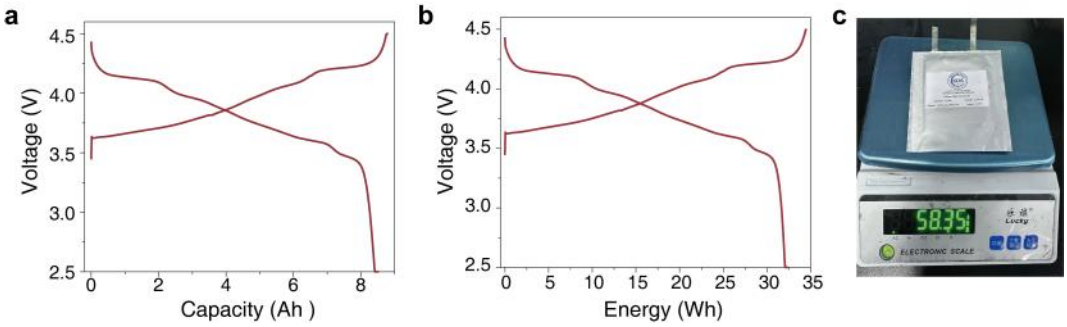

Figure S47. Electrochemical performance and photograph of 8.5 Ah LZNC||NCM pouch cell. a) Capacity-voltage, b) energy-voltage profiles and c) optical photograph of the 8.5 Ah LZNC||$LiNi_{0.98}Co_{0.01}Mn_{0.01}O_2$ pouch cell.

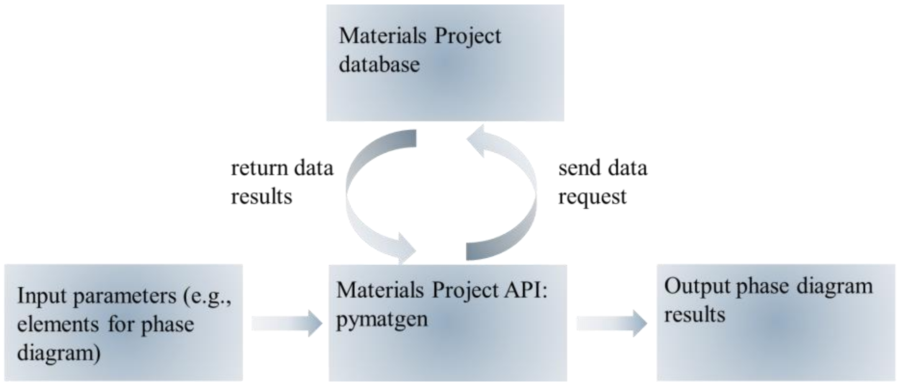

Figure S48.The workflow for phase diagram construction in this work.

Table S1. Lithiation reactions for Zn_3N_2. The energy values of the reactants and products are collected from the Materials Project website. The lower the reaction energy the more likely it is to occur, and it can be seen that LiZn and Li_3N are thermodynamically stable products

**Formula**	**Reaction energy (eV)**
$10Li + 5Zn_3N_2 \rightarrow 2Li_3N + 2LiN_3 + 2LiZnN + 13Zn$	-8.40
$Li + 3Zn \rightarrow LiZn_3$	-0.67
$Li + Zn \rightarrow LiZn$	-0.43

Table S2. Mass fractions of the components in the LZNC composite.

Component	Mass Fraction (%)	Description
LiZn	26.9%	Reaction product from Zn_3N_2 and Li
Li_3N	8.7%	Reaction product from Zn_3N_2 and Li
CNTs	16.7%	Introduced matrix
Excess Li	47.7%	Electrochemically active

Table S3. Comparison of volume and gravimetric specific capacity for reported composite negative electrode.

Composite material	Gravimetric specific capacity (mA h g^{-1})	Volume specific capacity (mA h cm^{-3})	Thickness (μm)	Ref.
$Li/Li_{22}Sn_5/LiF$	606	1599	104	[1]
Al-HCGB-Li	968	1776	50	[2]
Li/3D hollow carbon fiber	1107	363	165	[3]
$Li/Li_{22}Sn_5$	656	841	400	[4]
Li/hierarchical silver-nanowire–Graphene	1403	342	350	[5]
Li/CNTs-MC	1640	793	126	[6]
LiMg/CuCM	1401	633	104	[7]
Li/coralloid carbon fiber	1570	666	150	[8]
Li-B@SSM	996	1445	98.35	[9]
Fe-N@SSM-Li	628	571	105	[10]
3D $Li/Li_{22}Sn_5$	1178	1798	100	[11]
Li@GDD-CH	1598	652	46	[12]
ZOS-CF@Li	1115	584	137	[13]
TFA	1078	1552	100	[14]
This work	1799	1915	8	

Table S4. R_s and R_{ct} values obtained from EIS spectrum fitting for LZNC and lithium foil at different lithium plating/stripping stages under current densities of 1 mA cm^{-2} and 1 mA h cm^{-2}.

Cycle Number (n)	R_s of LZNC (Ω)	R_{ct} of LZNC (Ω)	Error of LZNC (%)	R_s of Li Foil (Ω)	R_{ct} of Li Foil (Ω)	Error of Li (%)
0	24.09	10.85	1.02	217	163.7	2.46
20	11.58	5.917	0.90	60.38	28.25	0.86
40	7.133	6.003	0.41	24.45	16.49	0.98
60	4.605	3.289	0.29	9.9	31.05	0.83
80	2.165	5.469	0.94	58.37	23.23	1.37
100	1.956	2.199	0.34	81.65	33.22	8.54

Table S5. The fitting data of Figure S26.

Temperature (K)	R_S of LZNC (Ω)	R_S of Li Foil (Ω)
293	4.01498	10.59
303	9.92	28.29
313	17.909	64.77
323	32.48	149.50
333	56.42	344.70

Table S6. Calculated specific energy of the 1A h LZNC||NCM811 pouch cell.

Composition	Parameter	Value
**NCM811 Positive electrode**	Active mass loading (mg cm^{-2})	40
	Areal capacity (mA h cm^{-2})	8
	Stacked number of layers	5
**LZNC Negative electrode**	Areal capacity (mA h cm^{-2})	14.87
	Thickness (μm)	80
	Areal weight (mg cm^{-2})	8.51
	Stacked number of layers	6
**Separator**	Areal weight (mg cm^{-2})	1
	Stacked number of layers	10
**Al Foil**	Areal weight (mg cm^{-2})	2.7
**Electrolyte**	Weight (mg)	1500
**Package foil**	Areal weight (mg cm^{-2})	23.1 (double layer)
**Cell**	Average Voltage (V)	3.8
	Capacity (A h)	0.99
	Mass (mg)	10310.94
	Gravimetric Specific energy (Wh kg^{-1})	364.85

Table S7. Comparison of cycling performance for previously reported pouch cells. (Total specific capacity of negative electrode refers to the ratio of the capacity of the cell exerted in cycling to the mass of the complete negative electrode including the collector.)

Modifications	Positive electrode	Rate	Cycling Life	Total Specific Capacity of Negative electrode	Capacity Decay Rate Per Cycle	Ref.
Li_2ZrF_6-based electrolytes	LFP	1C	220 cycles	230 mA h g^{-1}	0.091%	[15]
PFB electrolyte with a large oscillatory degree	NCM811	0.2C	150 cycles	554 mA h g^{-1}	0.053%	[16]
	NCM613	0.5C	400 cycles	681 mA h g^{-1}	0.049%	
Bilayer structure of SEI tailored through trioxane-modulated electrolytes	NCM811	0.2C	130 cycles	804 mA h g^{-1}	0.064%	[17]
A compatible electrolyte and uniform external pressure	NCM622	0.3C	200 cycles	635 mA h g^{-1}	0.070%	[18]
Solid-Solution Li-Ag alloy	NCM811	0.5C	250 cycles	337 mA h g^{-1}	0.052%	[19]
Anion-receptor-mediated carbonate electrolyte	NCM811	0.2C	50 cycles	570 mA h g^{-1}	0.182%	[20]
Li/C composite negative electrode	NCM523	0.1C	150 cycles	391 mA h g^{-1}	0.135%	[21]
Dual-Layered Artificial Interphase	NCM613	0.5C	60 cycles	391 mA h g^{-1}	0.152%	[22]
3D $Li/Li_{22}Sn_5$	NCM622	0.5C	45 cycles	295 mA h g^{-1}	0.207%	[11]
Tailoring solvation structures via precise diluent engineering	NCM811	1C	107 cycles	583 mA h g^{-1}	0.075%	[23]
Liquid–liquid interfacial tension	NCM811	0.1C	189 cycles	641 mA h g^{-1}	0.101%	[24]
This work	NCM811	0.5C	300 cycles	947 mA h g^{-1}	0.028%	

Table S8. Parameters of the 8.5A h LZNC||$LiNi_{0.98}Co_{0.01}Mn_{0.01}O_2$ pouch cell.

Composition	Parameter	Value
**Ni98 Positive electrode**	Areal capacity (mA h cm^{-2})	11.8
	Mass loading (mg cm^{-2})	27.5
	Stacked number of layers	15
**LZNC Negative electrode**	Areal capacity (mA h cm^{-2})	14.87
	Thickness (μm)	80
	Stacked number of layers	16
**Separator**	Areal weight (mg cm^{-2})	1
**Al Foil**	Thickness (μm)	12
**Electrolyte**	Volumes (g)	12.75
**Cell**	Energy (W h)	32.2924
	Mass (g)	58.35
	Gravimetric Specific energy (Wh kg^{-1})	553.43

References

1. Li, G. *et al.* Locking Active Li Metal through Localized Redistribution of Fluoride Enabling Stable Li-Metal Batteries. *Adv. Mater.* **35**, 2207310 (2023).
2. Shi, P. *et al.* Inhibiting intercrystalline reactions of negative electrode with electrolytes for long-cycling lithium batteries. *Sci. Adv.* **8**, eabq3445 (2022).
3. Liu, L. *et al.* Free-standing hollow carbon fibers as high-capacity containers for stable lithium metal negative electrodes. *Joule* **1**, 563–575 (2017).
4. Wan, M. *et al.* Mechanical rolling formation of interpenetrated lithium metal/lithium tin alloy foil for ultrahigh-rate battery negative electrode. *Nat. Commun.* **11**, 829 (2020).
5. Xue, P. *et al.* A hierarchical silver-nanowire–graphene host enabling ultrahigh rates and superior long-term cycling of lithium-metal composite negative electrodes. *Adv. Mater.* **30**, 1804165 (2018).
6. Xie, J. *et al.* Incorporating flexibility into stiffness: self-grown carbon nanotubes in melamine sponges enable a lithium-metal-negative electrode capacity of 15 mA h cm^{-2} Cyclable at 15 mA cm^{-2}. *Adv. Mater.* **31**, 1805654 (2019).
7. Luo, C. *et al.* Roll-to-roll fabrication of zero-volume-expansion lithium-composite negative electrodes to realize high-energy-density flexible and stable lithium-metal batteries. **34**, 2205677 (2022).
8. Zhang, R. *et al.* Coralloid carbon fiber-based composite lithium negative electrode for robust lithium metal batteries. *Joule* **2**, 764–777 (2018).
9. Qing, P. *et al.* Highly reversible lithium metal negative electrode enabled by 3D lithiophilic–lithiophobic dual-skeletons. *Adv. Mater.* **35**, 2211203 (2023).
10. Fu, X., Duan, H., Zhang, L., Hu, Y. & Deng, Y. A 3D Framework with an In Situ Generated Li3N Solid Electrolyte Interphase for Superior Lithium Metal Batteries. *Adv. Funct. Mater.* **33**, 2308022 (2023).
11. Liang, H. *et al.* Boosting the Intrinsic Stability of Lithium Metal Negative electrodes by an Electrochemically Active Encapsulating Framework. *Adv. Energy Mater.* **13**, 2302755 (2023).
12. Zhang, Y., Yao, M., Wang, T., Wu, H. & Zhang, Y. A 3D Hierarchical Host with Gradient-

Distributed Dielectric Properties toward Dendrite-free Lithium Metal Negative electrode. *Angew. Chem. Int. Ed.* **63**, e202403399 (2024).

13. Liu, X. *et al.* Constructing Fast Ion/Electron Conducting Pathway within 3D Stable Scaffold for Dendrite-Free Lithium Metal Negative electrode. *Adv. Funct. Mater.* **35**, 2420382 (2025).
14. Zhang, X. *et al.* Topology Fortified Negative electrodes Powered High-Energy All-Solid-State Lithium Batteries. *Adv. Mater.* **37**, 2506298 (2025).
15. Xu, Q. *et al.* Li_2ZrF_6-based electrolytes for durable lithium metal batteries. *Nature* **637**, 339–346 (2025).
16. Zhang, S. *et al.* Oscillatory solvation chemistry for a 500 Wh kg^{-1} Li-metal pouch cell. *Nat. Energy.* **9**, 1285–1296 (2024).
17. Zhang, Q.-K. *et al.* Homogeneous and mechanically stable solid–electrolyte interphase enabled by trioxane-modulated electrolytes for lithium metal batteries. *Nat. Energy.* **8**, 725–735 (2023).
18. Niu, C. *et al.* High-energy lithium metal pouch cells with limited negative electrode swelling and long stable cycles. *Nat. Energy.* **4**, 551–559 (2019).
19. Ye, Y. *et al.* Solid-solution or intermetallic compounds: phase dependence of the Li-alloying reactions for Li-metal batteries. *J. Am. Chem. Soc.* **145**, 24775–24784 (2023).
20. Huang, K. *et al.* Regulation of SEI formation by anion receptors to achieve ultra-stable lithium-metal batteries. *Angew. Chem. Int. Ed.* **60**, 19232–19240 (2021).
21. Shi, P. *et al.* A Successive conversion-deintercalation delithiation mechanism for practical composite lithium negative electrodes. *J. Am. Chem. Soc.* **144**, 212–218 (2022).
22. Guo, J. *et al.* A self-Reconfigured, dual-Layered artificial interphase toward high-current-density quasi-solid-state lithium metal batteries. *Adv. Mater.* **35**, 2300350 (2023).
23. Peng, J. *et al.* Tailoring Solvation Structures via Precise Diluent Engineering for High-Rate 500 Wh kg−1 Lithium-Metal Batteries. *Adv. Mater.* **n/a**, e09109.
24. Ji, H. *et al.* Liquid–liquid interfacial tension stabilized Li-metal batteries. *Nature* **643**, 1255–1262 (2025).
