# Supplementary material for: Engineering thin 3D Li-composite foil negative electrodes with high mechanical toughness
Source: Nat Commun. 2026 Feb 4;17:2345. doi: 10.1038/s41467-026-69155-z (PMC12979835; doi:10.1038/s41467-026-69155-z)
Supplement: Supplementary file 2 — Description of Additional Supplementary Files [file 41467_2026_69155_MOESM2_ESM.pdf]

Description of Additional Supplementary Files

Supplementary Data 1. The source files of MD simulation structure.
