# Supplementary figures and images for: Engineering thin 3D Li-composite foil negative electrodes with high mechanical toughness
Source: Nat Commun. 2026 Feb 4;17:2345. doi: 10.1038/s41467-026-69155-z (PMC12979835; doi:10.1038/s41467-026-69155-z)

Initial configuration
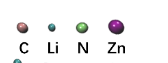


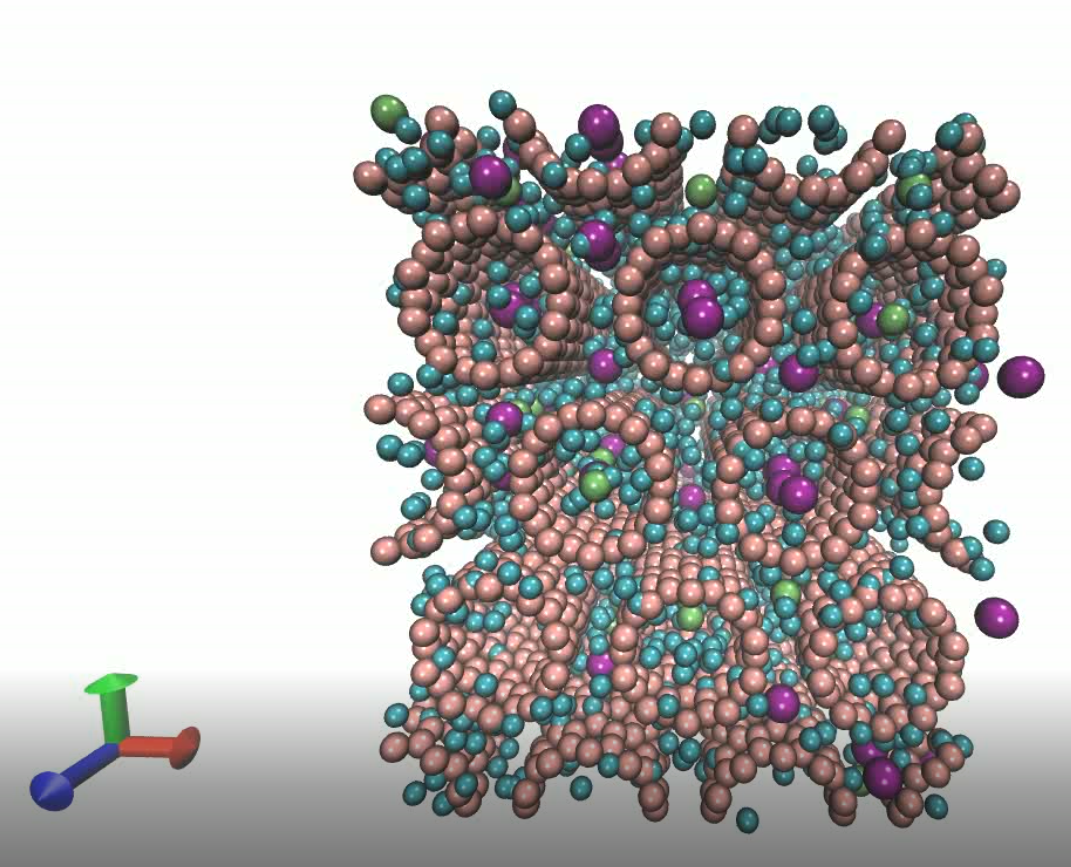


Final configuration


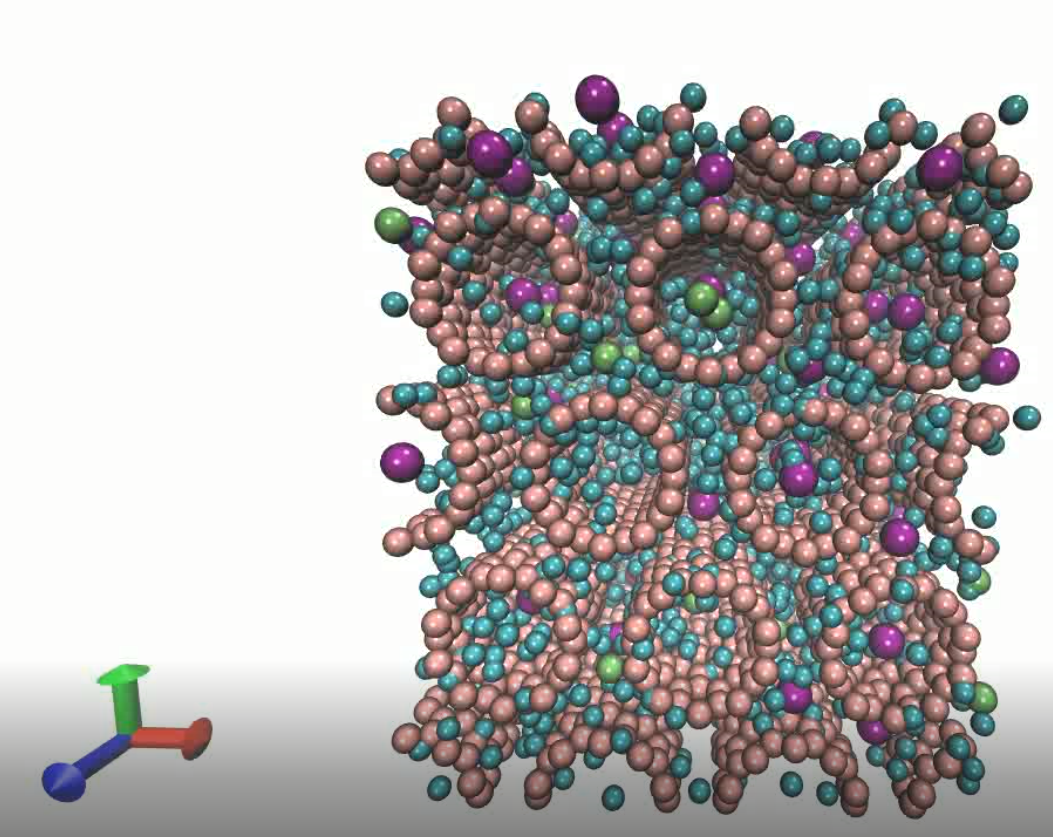

Supplement: Supplementary file 3 — Supplementary Data 1 [file 41467_2026_69155_MOESM3_ESM.zip › Supplementary Data 1/MD simulation/Initial and final configuration.docx]
